# Synthesis of (1*E*,3*E*)-1,4-diarylbuta-1,3-dienes promoted by μ-OMs palladium–dimer complex

**DOI:** 10.1186/s13065-019-0561-3

**Published:** 2019-03-28

**Authors:** Xueliang Zhou, Yuan Zhou, Qiang Zhu, Huimin Chen, Nan Wu, Xiangru Wen, Zhou Xu

**Affiliations:** School of Pharmacy, Xuzhou Medical University, Xuzhou, Jiangsu Province China

**Keywords:** μ-OMs palladium–dimer, (1*E*,3*E*)-1,4-Diarylbuta-1,3-dienes, β-Bromostyrene, Olefin, Fluorescence

## Abstract

A convenient approach for (1*E*,3*E*)-1,4-diarylbuta-1,3-dienes via intermolecular Heck reaction of olefins and β-bromostyrenes promoted by μ-OMs palladium–dimer complex has been successfully developed. The products 1,4-conjugated dienes could be obtained with good yield (up to 82%). The catalyst system has excellent chemical selectivity and group tolerance which could supply an alternative way to gain the valuable biaryl linkage building blocks. Furthermore, fluorescence studies of dienes showed that some of them may have potential applications as luminescent clusters.

**Electronic supplementary material:**

The online version of this article (10.1186/s13065-019-0561-3) contains supplementary material, which is available to authorized users.

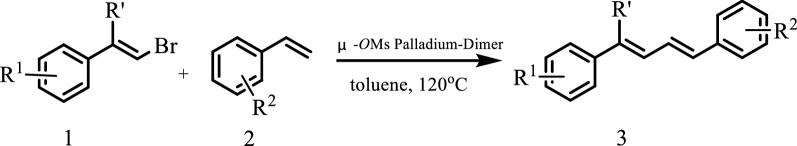

## Introduction

**Fig. 1:**
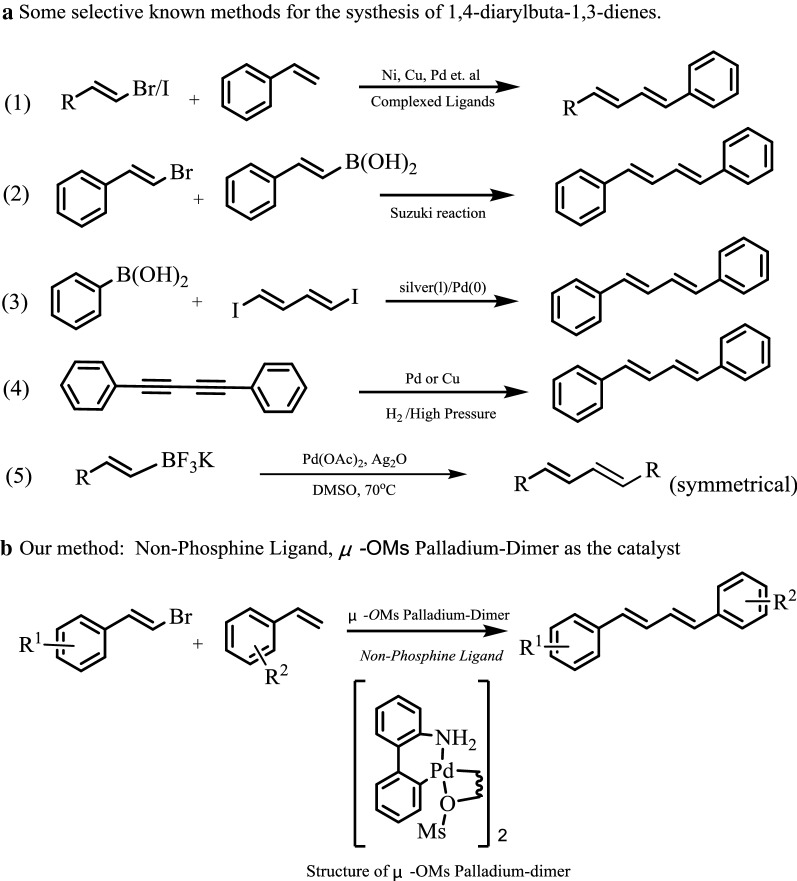
Representative synthetic strategies for 1,4-diarylbuta-1,3-dienes and our approach

1,4-Diarylbuta-1,3-dienes are found not only as the important building blocks in polymers, but also in variety of synthetic/naturally occurring biologically active molecules, which possess a wide range of bioactivities [1–4]. Several methods have been developed for the synthesis of 1,3-dienes via cross-coupling reaction catalyzed by transition metal catalysts, such as Ni [5, 6], Cu [7, 8] and Pd [9, 10] (Fig. 1, equation 1). Some alternative methods were also reported. For example, 1,3-dienes could also be achieved via the Suzuki–Miyaura reaction between vinyl boric acid and vinyl bromides [11, 12] (Fig. 1, equation 2). The coupling of (1*E*,3*E*)-1,4-diiodobuta-1,3-diene with arylboronic reagents was reported for the synthesis of symmetrical 1,4-diarylbuta-1,3-dienes [13] (Fig. 1, equation 3). Controlled hydrogenation of the triple bond of 1,2-diphenylacetylenes under high pressure and temperature could lead to the formation of stilbenes without the formation of new C–C bond [14, 15] (Fig. 1, equation 4). Homocoupling of potassium alkenyltrifluoroborates is an effective method for the synthesis of symmetrical 1,3-dienes [16] (Fig. 1, equation 5). However, despite these major advances, discovery of new catalyst system for the construction of 1,3-diene units (both symmetrical and unsymmetrical ones) with good chemical selectivity and easy gained catalyst is still an attractive goal.

Heck reactions is one of the most important reaction among transition metal catalyzed C–C bond formation methods and many important catalyst systems have been achieved. However, most of the results which have been described with Pd catalysts, were obtained for the coupling of aryl halides [17–21]. Relatively few results have been reported with vinyl halides. The researchers have put more attentions to improve the Heck reaction conditions and catalysts such as immobilized catalyst, fluorine chemical, no phosphorus catalyst, et al. [22–25]. μ-OMs palladium–dimer was first reported by Buchwald, which was used as Pd-precatalyst for C–N/C–C coupling reactions [26]. Although, the μ-OMs dimer is very easy to obtain and always used as the optimal palladium source in many reactions, it has not been used directly as the catalyst for Heck reaction. For the long run, our research interests focus on the studies on noble metal salts catalyzed reactions [27–32]. As our continuous research interest, herein, we report that Pd–dimer (namely μ-OMs dimer) which is a typically non-phosphorus Buchwald Pd-precatalyst could be successfully applied in the Heck reaction of olefins and β-bromostyrenes affording 1,4-conjugated dienes with good yield and excellent chemical selectivity (Fig. 1b).

## Results and discussion

To begin, we chose the Heck reaction between (*E*)-(2-bromovinyl)benzene (**1a**) and styrene (**2a**) in the presence of Pd(OAc)_2_ as the model system to optimize the reaction conditions for the synthesis of the product **3a**. In an initial experiment, the reaction was performed in toluene at 80 °C catalyzed by 5 mol% Pd(OAc)_2_ with K_2_CO_3_ as the base, isolating (1*E*,3*E*)-1,4-diphenylbuta-1,3-diene (**3a**) in 19% yield after 24 h (Table 1, entry 1). Then, we investigated different palladium catalysts on the reaction yield and catalytic efficiency. As depicted in Table 1, when Pd_2_(dba)_3_ or Pd(TFA)_2_ was used in place of Pd(OAc)_2_, similar results were achieved (Table 1, entries 2–3). The homemade dimer palladium catalyst was then examined, and the results showed that its catalytic activity was superior to other palladium catalysts (entries 4 vs 1–3). To our surprise, when the coupling reaction was carried with traditional Xphos or PPh_3_ as supporting ligands and with μ-OMs dimer as palladium source, the results clearly indicated that both XPhos and PPh_3_ were the poor ligands for this transformation (Table 1, entries 5–6). When the reaction temperature was increased to 120 °C, the yield of the product improved significantly and the reaction time was also shortened (Table 1, entry 7).

**Table 1:** Optimization for the reaction condition
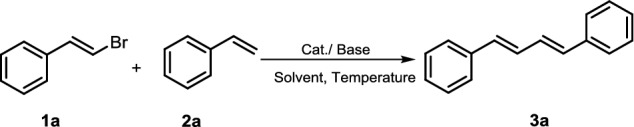

Entry	Catalyst/5%	Solvent	Base	T/°C	t/h	Yield/%^a^
1	Pd(OAc)_2_	Toluene	K_2_CO_3_	80	36	19
2	Pd_2_(dba)_3_	Toluene	K_2_CO_3_	80	24	11
3	Pd(OAcCF_3_)_2_	Toluene	K_2_CO_3_	80	24	15
4	μ-OMs dimer	Toluene	K_2_CO_3_	80	36	51
5^b^	μ-OMs dimer/PPh_3_	Toluene	K_2_CO_3_	80	24	Trace
6	Pd-Xphos	Toluene	K_2_CO_3_	80	24	Mix
7	μ-OMs dimer	Toluene	K_2_CO_3_	120	18	74
8	μ-OMs dimer	DMF	K_2_CO_3_	120	12	52
9	μ-OMs dimer	CH_3_CN	K_2_CO_3_	120	12	21
10	μ-OMs dimer	NMP	K_2_CO_3_	120	12	50
11	μ-OMs dimer	DMSO	K_2_CO_3_	120	12	48
12	μ-OMs dimer	Toluene	Li_2_CO_3_	120	24	mix
13	μ-OMs dimer	Toluene	Cs_2_CO_3_	120	15	68
14	μ-OMs dimer	Toluene	K_3_PO_4_	120	20	62
15	μ-OMs dimer	Toluene	Et_3_N	120	18	70
16^c^	μ-OMs dimer	Toluene	K_2_CO_3_	120	24	65

Reactions were carried under Ar in Schlenk flasks. **1a** (0.5 mmol), **2a** (0.5 mmol), Pd* (5 mol%), solvent (2 mL), base (2.0 equiv.), 80–120 °C

^a^Isolated yields

^b^Reaction condition: μ-OMs dimer (0.01 mmol)/PPh_3_ (0.011 mol) was stirred at room temperature in DCM (1 mL) under Ar for 0.5 h, then **1a** (0.5 mmol), **2a** (0.5 mmol), toluene (2 mL), K_2_CO_3_ (2.0 equiv.) were added and stirred at 80 °C

^c^The catalyst loading is 2.5 mol%

Subsequently, different solvents were examined using μ-OMs Pd–dimer as the catalyst, in the presence of K_2_CO_3_ under an argon atmosphere. In a series of reaction solvent, we found that toluene as low polar solvent was the best one among the solvents screened. Polar aprotic solvents, such as DMF, NMP and DMSO gave almost the same level yield, while CH_3_CN gave much poor yield (Table 1, entries 8–11). Thus, toluene was used as the best solvent for further studies. Finally, we investigated the effect of bases on the reaction, including K_3_PO_4_, Li_2_CO_3_, Cs_2_CO_3_ and Et_3_N. Of the base screened, the best result was obtained with K_2_CO_3_ which could make the reaction faster and more efficiency (entry 6). Cs_2_CO_3_, K_3_PO_4_ and Et_3_N gave slightly inferior yield than K_2_CO_3_ (entries 13–15), while using Li_2_CO_3_ as the base gave complexed mixture which might due to the strong basicity of Li_2_CO_3_. The yield was reduced by reducing the amount of catalyst from 5 to 2.5% (Table 1, entry 16).

Under the above optimized reaction conditions, we explored the generality and applicability of the protocol. Firstly, the reaction between different styrenes and (*E*)-(2-bromovinyl)benzene was examined. When chlorine was in the ortho, meta- or para-position of benzene ring in styrene substrates, the para position substrate gave the highest yield of the corresponding product with 82%, while the ortho-position substrate gave the lowest yield which might due to the steric effect (Table 2, entries 1–3). Styrene with electron-donating group on the benzene ring, such as methyl group on the para- position, gave lower yield than that of electron withdrawing one (Table 2, entry 1 vs 4). A wide variety of vinyl bromides, bearing either electron-donating or electron-withdrawing substituents, were successfully coupled with styrene partner with 54–68% yield. Generally, electron-withdrawing groups have positive impacts on the building of conjugated dienes, compared with electronic-donating ones (Table 2, entries 5, 7, 11 vs 6, 8–9). Unfortunately, when R^1^ group was 4-Br, the reaction gave a messy mixture which may due to the different active reaction sites of the vinyl bromide (Table 2, entry 10). Interestingly, when R^2^ group was 4-Br, the reaction could proceed smoothly and afforded the corresponding product with 61% yield (Table 2, entry 16). The substituent on the aryl ring of β-bromostyrene such as a para-fluride, a para-cholor group decreased slightly the reaction rates and the yields (Table 2, entry 1 vs 12–13). An electron-donating substituent, such as a para-methyl group, on the aryl ring of β-bromostyrene could afford better yields than electron-withdrawing ones (Table 2, entries 12–13 vs 15, 2 vs 14).

**Table 2:** Scope of the reaction
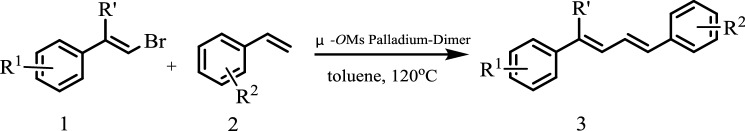

Entry^a^	R^1^	R^2^	Product	t/h	Yield/%^b^
1	H	4-Cl	**3b**	18	82
2	H	3-Cl	**3c**	18	61
3	H	2-Cl	**3d**	24	58
4	H	4-CH_3_	**3e**	18	68
5	4-Cl	H	**3b**	24	75
6	4-OCH_3_	H	**3f**	36	55
7	4-F	H	**3g**	18	82
8	4-CH_3_	H	**3e**	18	68
9	4-OCOCH_3_	H	**3h**	30	54
10	4-Br	H	**–**	24	Mixture
11	4-Cl, R′ = Me	H	**3i**	18	65
12	4-F	4-Cl	**3j**	24	71
13	4-Cl	4-Cl	**3k**	24	63
14	4-CH_3_	3-Cl	**3l**	18	66
15	4-CH_3_	4-Cl	**3m**	20	74
16	4-F	4-Br	**3n**	24	61

The reactions were carried in Schlenk flask under Ar. Olefins (0.5 mmol), β-bromostyrenes (0.5 mmol), μ-OMs dimer–Pd (5 mol%), toluene (2 mL), k_2_CO_3_ (2.0 equiv.), 120 °C

^a^For entry 11, R′ = Me, for other entries, R′ = H

^b^Isolated yield

**Fig. 2:**
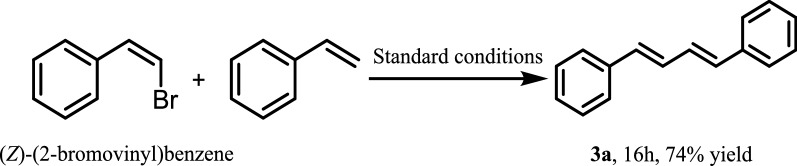
Chemical selectivity of the reaction

Interestingly, when the reaction between (Z)-(2-bromovinyl) benzene and styrene was examined, only (1*E*,3*E*)-diene was obtained which indicated that our catalyst system had excellent chemical selectivity (Fig. 2) [33].

**Fig. 3:**
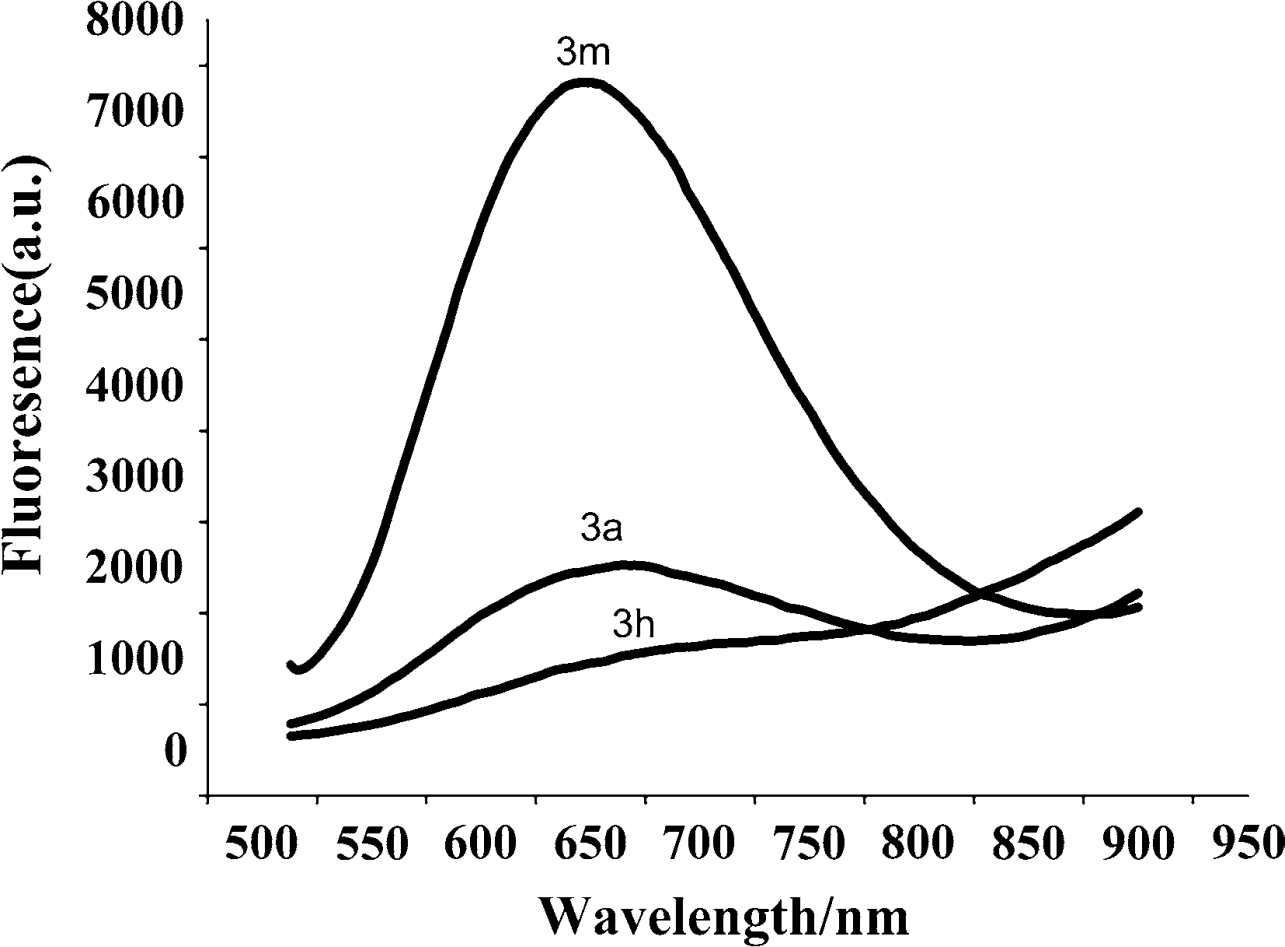
Fluorescence spectra of 3a, 3e and 3m (concentration: 10 μmol/L) upon excitation at 331 nm, 228 nm and 237 nm respectively. Samples were dissolved in DMSO/PBS (30%)

To broaden the possible usage of these compounds, the fluorescence activity of **3a**, **3m** and **3e** was studied. As can be seen from Fig. 3, **3a** and **3m** showed fluorescence, and the excitation wavelength was 650 nm and 657 nm respectively, which reach the near infrared region. They may have potential applications as fluorescent materials. While at the same concentration, **3e** has no fluorescence.

## Conclusions

In summary, we have successfully developed an approach for the synthesis of (1*E*,3*E*)-1,4-diarylbuta-1,3-dienes via intermolecular Heck reaction of olefins and β-bromostyrenes promoted by μ-OMs palladium–dimer complex catalyst. The products 1,4-conjugated dienes could be obtained with good yield (up to 82%). The catalyst system has good chemical selectivity and excellent group tolerance which would supply an alternative way to gain the valuable biaryl linkage building blocks.

## Experimental

### General information

Commercially available reagents were used without further purification. The solvents used for experiment research were all through pretreatment on condition of anaerobic and without water. Reactions were monitored by TLC using Silicycle precoated silica gel plates. Flash column chromatography was performed over Silicycle silica gel (300–400 mesh). ^1^H NMR and ^13^C NMR spectra were recorded on JMTC-400/54/SS 400 MHz spectrometers using residue solvent peaks as internal standards (CHCl_3_, ^1^H: 7.26 ppm; ^13^C: 77.00 ppm). The fluorescence spectra of samples were detected with a Fluorescence spectrophotometer (F-4600FL Spectrophotometer, Hitachi, Japan) using a Xenon lamp as the excitation source at room temperature, and the excitation wavelength was 331 nm, 228 nm and 237 nm.

### General procedure for the synthesis of 1,4-diarylbuta-1,3-dienes

A Schlenk tube was charged with styrene (20.8 mg, 0.2 mmol), (*E*)-(2-bromovinyl) benzene (36.6 mg, 2.0 mmol), μ-OMs dimer (7.3 mg, 5% mmol), K_2_CO_3_ (55.3 mg, 2.0 mmol), and anhydrous toluene 1.0 mL under an Ar atmosphere. The Schlenk tube was sealed with a Teflon valve, and then the reaction mixture was stirred at 110 °C for 24 h, monitoring by TLC. After the reaction was completed, the reaction mixture was extracted with ethyl acetate three times. Then the filtrates were dried over Na_2_SO_4_ and concentrated under reduced pressure. The residue obtained was purified by chromatography (silica gel, PE–EtOAc, 100:1) to give the product.

### Product characterization data

#### (1*E*,3*E*)-1,4-diphenylbuta-1,3-diene (**3a**) [34–36]

The product was obtained as white solid in 74% yield. ^1^H NMR (400 MHz, CDCl_3_) δ 7.44 (d, *J* = 7.4 Hz, 4H), 7.33 (t, *J* = 7.8 Hz, 4H), 7.24 (d, *J* = 5.6 Hz, 2H), 6.96 (dd, *J *= 12.0, 2.6 Hz, 2H), 6.67 (dd, *J* = 12.0, 2.6 Hz, 2H); ^13^C NMR (100 MHz, CDCl_3_) δ 137.3, 132.8, 129.2, 128.6, 127.5, 126.4 (see Additional file 1).

#### 1-Chloro-4-((1*E*,3*E*)-4-phenylbuta-1,3-dien-1-yl)benzene (**3b**) [37]

The product was obtained as white solid in 82% yield. ^1^H NMR (400 MHz, CDCl_3_) δ 7.43 (d, *J* = 7.4 Hz, 2H), 7.39–7.20 (m, 7H), 7.02–6.84 (m, 2H), 6.75–6.53 (m, 2H); ^13^C NMR (100 MHz, CDCl_3_) δ 137.3, 136.0, 133.5, 131.5, 130.0, 129.0, 128.9, 128.8, 127.8, 127.6, 126.5 (see Additional file 1).

#### 1-Chloro-3-((1*E*,3*E*)-4-phenylbuta-1,3-dien-1-yl)benzene (**3c**) [38]

The product was obtained as white solid in 61% yield. ^1^H NMR (400 MHz, CDCl_3_) δ 7.49–7.39 (m, 3H), 7.33 (t, *J* = 7.5 Hz, 2H), 7.29 (dt, *J *= 7.6, 1.5 Hz, 1H), 7.27–7.21 (m, 4H), 7.19 (dt, *J* = 7.6, 1.6 Hz, 1H), 7.00–6.85 (m, 2H), 6.78–6.64 (m, 1H), 6.64–6.53 (m, 1H); ^13^C NMR (100 MHz, CDCl_3_) δ 139.3, 137.1, 134.6, 133.9, 131.1, 130.6, 129.8, 128.7, 128.7, 127.8, 127.4, 126.5, 126.1, 124.5 (see Additional file 1).

#### 1-Chloro-2-((1*E*,3*E*)-4-phenylbuta-1,3-dien-1-yl)benzene (**3d**) [39]

The product was obtained as white solid in 58% yield. ^1^H NMR (400 MHz, CDCl_3_) δ 7.62 (dd, *J* = 7.8, 1.4 Hz, 1H), 7.45 (d, *J* = 7.3 Hz, 2H), 7.39–7.30 (m, 3H), 7.29–7.20 (m, 4H), 7.15 (td, *J* = 7.7, 1.5 Hz, 1H), 7.11–6.88 (m, 3H), 6.71 (d, *J *= 15.1 Hz, 1H); ^13^C NMR (100 MHz, CDCl_3_) δ 137.1, 135.3, 133.9, 133.2, 131.6, 129.9, 129.1, 128.7, 128.4, 128.4, 127.8, 126.8, 126.2 (see Additional file 1).

#### 1-Methyl-4-((1*E*,3*E*)-4-phenylbuta-1,3-dien-1-yl)benzene (**3e**) [40, 41]

The product was obtained as white solid in 68% yield. ^1^H NMR (400 MHz, CDCl_3_) δ 7.49–7.39 (m, 2H), 7.38–7.27 (m, 4H), 7.24–7.18 (m, 1H), 7.13 (d, *J* = 8.2 Hz, 2H), 7.01–6.80 (m, 2H), 6.64 (td, *J* = 10.7, 6.6 Hz, 2H), 2.34 (s, 3H); ^13^C NMR (100 MHz, CDCl_3_) δ 137.6, 137.6, 134.7, 132.9, 132.3, 129.5, 129.5, 128.7, 128.4, 127.5, 126.4, 21.4 (see Additional file 1).

#### 1-Methoxy-4-((1*E*,3*E*)-4-phenylbuta-1,3-dien-1-yl)benzene (**3f**) [42]

The product was obtained as white solid in 55% yield. ^1^H NMR (400 MHz, CDCl_3_) δ 7.46–7.35 (m, 4H), 7.31 (t, *J* = 7.5 Hz, 2H), 7.23–7.17 (m, 1H), 7.01–6.73 (m, 4H), 6.61 (d, *J* = 15.1 Hz, 2H), 3.81 (s, 3H); ^13^C NMR (100 MHz, CDCl_3_) δ 159.3, 137.5, 132.4, 131.7, 129.5, 128.6, 127.6, 126.2, 114.1, 55.3 (see Additional file 1).

#### 1-Fluoro-4-((1*E*,3*E*)-4-phenylbuta-1,3-dien-1-yl)benzene (**3g**) [41]

The product was obtained as white solid in 82% yield. ^1^H NMR (400 MHz, CDCl_3_) δ 7.50–7.37 (m, 4H), 7.33 (t, *J* = 7.5 Hz, 2H), 7.25–7.20 (m, 1H), 7.09–6.99 (m, 2H), 6.90 (qd, *J* = 14.9, 10.5 Hz, 2H), 6.65 (t, *J* = 14.4 Hz, 2H); ^13^C NMR (100 MHz, CDCl_3_) δ 163.7, 135.9, 133.5 (d, J = 3.8 Hz), 133.2, 132.2, 131.5, 129.7, 128.9, 128.7 (d, *J* = 1.9 Hz), 128.0 (d, *J *= 8.6 Hz), 127.6, 115.8 (d, *J* = 21.0 Hz) (see Additional file 1).

#### 4-((1*E*,3*E*)-4-phenylbuta-1,3-dien-1-yl)phenylacetate (**3h**)

The product was obtained as white solid in 54% yield. ^1^H NMR (400 MHz, CDCl_3_) δ 7.43 (d, J = 8.7 Hz, 4H), 7.37–7.29 (m, 2H), 7.24–7.17 (m, 1H), 7.05 (dt, *J* = 9.3, 2.3 Hz, 2H), 7.00–6.78 (m, 2H), 6.72–6.59 (m, 2H), 2.33–2.24 (3H); ^13^C NMR (100 MHz, CDCl_3_) δ 169.5, 150.0, 137.3, 135.2, 133.0, 131.7, 129.5, 129.1, 128.7, 127.6, 127.3, 126.4, 121.8, 21.2; IR (cm^−1^): 3043, 2994, 2990, 1772, 1665, 1502, 1480, 1111, 990; HRMS ESI–TOF: m/z = 265.1134 [M+H]^+^ (265.1129 calcd for C_18_H_17_O_2_) (see Additional file 1).

#### 1-Chloro-4-((2*E*,4*E*)-5-phenylpenta-2,4-dien-2-yl)benzene (**3i**) [43]

The product was obtained as white solid in 65% yield. ^1^H NMR (400 MHz, CDCl_3_) δ 7.53–7.38 (m, 4H), 7.37–7.27 (m, 4H), 7.24–7.03 (m, 2H), 6.77–6.55 (m, 2H), 2.21 (s, 3H); ^13^C NMR (100 MHz, CDCl_3_) δ 141.3, 137.5, 135.4, 133.4, 132.8, 128.7, 128.4, 127.7, 127.6, 126.8, 126.4, 125.5, 16.1 (see Additional file 1).

#### 1-Chloro-4-((1*E*,3*E*)-4-(4-fluorophenyl)buta-1,3-dien-1-yl) benzene (**3j**) [44]

The product was obtained as white solid in 71% yield. ^1^H NMR (400 MHz, CDCl_3_) δ 7.48–7.34 (m, 4H), 7.33–7.27 (m, 2H), 7.03 (t, *J* = 7.8 Hz, 2H), 6.97–6.78 (m, 2H), 6.62 (t, *J* = 14.6 Hz, 2H); ^13^C NMR (100 MHz, CDCl_3_) δ 135.8, 133.4, 133.4, 132.1, 131.4, 129.6, 128.8, 127.9, 127.9, 115.8, 115.6 (see Additional file 1).

#### (1*E*,3*E*)-1,4-bis(4-chlorophenyl)buta-1,3-diene (**3k**) [45, 46]

The product was obtained as white solid in 63% yield. ^1^H NMR (400 MHz, CDCl_3_) δ 7.42–7.33 (m, 4H), 7.33–7.27 (m, 4H), 6.96–6.83 (m, 2H), 6.69–6.57 (m, 2H); ^13^C NMR (100 MHz, CDCl_3_) δ 135.7, 133.2, 131.9, 129.5, 128.9, 127.5 (see Additional file 1).

#### 1-Chloro-3-((1*E*,3*E*)-4-(p-tolyl)buta-1,3-dien-1-yl)benzene (**3l**)

The product was obtained as white solid in 66% yield. ^1^H NMR (400 MHz, CDCl_3_) δ 7.46–7.41 (m, 1H), 7.36 (d, *J *= 8.2 Hz, 2H), 7.32–7.24 (m, 2H), 7.20 (dt, *J* = 7.6, 1.7 Hz, 1H), 7.16 (d, *J *= 7.8 Hz, 2H), 7.02–6.82 (m, 2H), 6.69 (d, *J* = 14.6 Hz, 1H), 6.58 (d, *J* = 14.6 Hz, 1H), 2.37 (s, 3H); ^13^C NMR (100 MHz, CDCl_3_) δ 139.4, 137.8, 134.6, 134.3, 133.9, 130.8, 130.5, 129.8, 129.4, 127.8, 127.2, 126.4, 126.1, 124.5; IR (cm^−1^): 3033, 2990, 2984, 1768, 1640, 1512, 1486, 1123; HRMS ESI-TOF: m/z = 255.0941 [M+H]^+^ (255.0947 calcd for C_17_H_16_Cl) (see Additional file 1).

#### 1-Chloro-4-((1*E*,3*E*)-4-(p-tolyl)buta-1,3-dien-1-yl)benzene (**3m**) [44]

The product was obtained as white solid in 74% yield. ^1^H NMR (400 MHz, CDCl_3_) δ 7.43–7.33 (m, 4H), 7.33–7.29 (m, 2H), 7.16 (d, J = 7.8 Hz, 2H), 7.04–6.80 (m, 2H), 6.78–6.51 (m, 2H), 2.37 (s, 3H); ^13^C NMR (100 MHz, CDCl_3_) δ 137.7, 136.0, 134.4, 133.4, 132.9, 130.8, 130.0, 129.4, 128.8, 127.9, 127.4, 126.4, 21.3 (see Additional file 1).

#### 1-Bromo-4-((1*E*,3*E*)-4-(4-fluorophenyl)buta-1,3-dien-1-yl)benzene (**3n**) [45, 46]

The product was obtained as white solid in 61% yield. ^1^H NMR (400 MHz, CDCl_3_) δ 7.53–7.35 (m, 4H), 7.29 (d, *J *= 8.2 Hz, 2H), 7.03 (t, *J* = 8.7 Hz, 2H), 6.96–6.77 (m, 2H), 6.73–6.53 (m, 2H); ^13^C NMR (100 MHz, CDCl_3_) δ 163.7, 136.5, 133.7 (d, *J* = 2.8 Hz), 132.5, 132.1, 131.7, 130.0, 128.9 (d, *J* = 1.9 Hz), 128.2 (d, *J *= 7.8 Hz), 128.1, 121.6, 115.9 (d, *J* = 20.0 Hz) (see Additional file 1).

## Additional file

**Additional file 1.** The synthesis of starting materials, general procedure for the products and ^1^H-NMR and ^13^C-NMR spectra of all products.

## Abbreviations

DMF: dimethyl formamide
NMP: *N*-methyl pyrrolidone
DMSO: dimethyl sulfoxide
Ar: argon gas
PBS: phosphate buffer saline
TLC: thin layer chromatography
PE: petroleum ether
