# Supplementary material for: Synthesis of (1E,3E)-1,4-diarylbuta-1,3-dienes promoted by μ-OMs palladium–dimer complex
Source: BMC Chem. 2019 Mar 28;13(1):39. doi: 10.1186/s13065-019-0561-3 (PMC6661770; doi:10.1186/s13065-019-0561-3)
Supplement: Supplementary file 1 — Additional file 1. The synthesis of starting materials, general procedure for the products and 1H-NMR and 13C-NMR spectra of all products. [file 13065_2019_561_MOESM1_ESM.pdf]

# Supporting Information

## Efficient Synthesis of (1E, 3E)-1,4-Diarylbuta-1,3-Dienes Promoted by μ-OMs Palladium-Dimer Complex

Zhou Xueliang, Zhou Yuan, Zhu Qiang, Chen Huimin, Wu Nan, Wen Xiangru, Xu Zhou *

Jiangsu Key Laboratory of New Drug Research and Clinical Pharmacy, Xuzhou Medical University, Xuzhou 221004, China

*The authors requests for materials should be addressed to Z. Xu (email: xuzhou@xzhmu.edu.cn)

## Table of contents

I. General information........................................................................................2

II. Synthesis of starting materials and General procedure for the products................ 2

III. Fluorescence spectra of **3a**, **3e** and **3m**.................................................................... 3

IV. References ..........................................................................................................4

V. $^{1}H$ and $^{13}C$ NMR spectra of the synthetic compounds......................................... 4

## I. General information

General information: Unless otherwise noted, Commercially available reagents were used without further purification. all commercial materials were used without further purification. Methanol and 1,2-dichloroethane are ACS reagents grade, and were used as received unless otherwise noted. HFIP was purchased from Acros without further purification. TLC analysis of reaction mixtures was performed on silica gel GF254 TLC plates. Flash chromatography was carried out on Shanghai Titan technology co., LTD silica gel(300-400 mesh). $^1$H NMR, $^{13}$C NMR spectra were recorded with JEOL JMTC-400/54/SS 400 MHz spectrometers and using residue solvent peaks as internal standards.

## II. Synthesis of starting materials

### 1. General procedure for the preparation of (E)-(2-bromovinyl)benzene 1a[1]

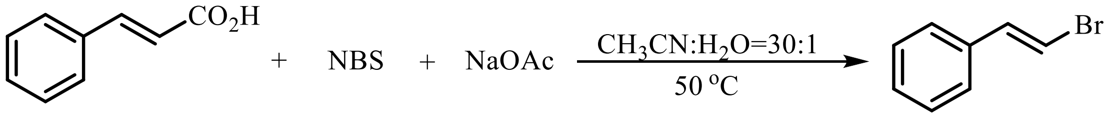


**(*E*)-S1** **1a**

Cinnamic acid (E)-S1 (2.96 g, 20 mmol), NBS (3.92 g,22 mmol), sodium acetate (0.33 g, 4 mmol) were added in a 100 ml round bottom flask containing 30 mL $CH_3CN$:$H_2O$ =30 : 1. The reaction mixture was stirred for 5-12 hours at 50 ℃. After the reaction was completed, cooled to room temperature and the reaction mixture was concentrated in *vacuo*, extracted with ethyl acetate. Then the combined organic layers were dried over $Na_2SO_4$ and the solution were concentrated under reduced pressure. The residue was purified through flash column chromatography on silica gel with petroleum ether and ethyl acetate (eluent: petroleum ether/ethyl acetate =10/1) to give the product.

### 2. Synthesis of (Z)-(2-bromovinyl)benzene[2,3]

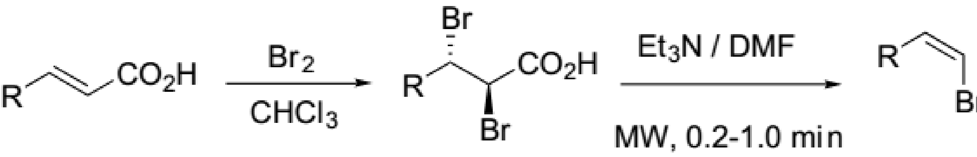


(*E*)-**1** **2** (*Z*)-**3**

Cinnamic acid (E) (2.96 g, 20 mmol), equal molar ratio $Br_2$ (20 mmol, 1.60 g) were added in a 50 ml round bottom flask containing 30 mL $CHCl_3$, The reaction mixture was stirred for the color fade at ice-bath. After the reaction was completed, the reaction mixture was concentrated under reduced pressure for removing chloroform and bromide. The solid was washed with water and dried over directly afford intermediate product 2.

A mixture of before preparation of the compound 2 (1 mmol) and triethylamine (1.05 mmol) was added to 2 ml DMF. The mixture was kept inside a microwave oven operated at 2450 MHz (TOSHIBA, ER-V11, 200 W) and was irradiated for 0.2–1.0 min without any stirring. The reaction mixture was then removed from the oven and cooled to room temperature. Water and ether were added to the reaction mixture and the organic layer was separated. Aqueous layer was extracted with ether. The combined organic layers were washed with water and brine, and dried over anhydrous magnesium sulfate. After evaporation of the solvent, the crude product was purified by column chromatography on silica gel with EtOAc–hexane to give (Z)-(2-bromovinyl)benzene.

### 3. Heck reaction: A typical procedure A

A Schlenk tube was charged with styrene (20.8 mg, 0.2 mmol), (E)-(2-bromovinyl) benzene (36.6 mg, 2.0 mmol), u-OMs Dimer (7.3 mg, 5% mmol), $K_2CO_3$ (55.3 mg, 2.0 mmol), and anhydrous Toluene 1.0 mL under a $N_2$ atmosphere. The Schlenk tube was sealed with a Teflon valve, and then the reaction mixture was stirred at 110 ℃ for 24 h, monitoring by TLC. After the reaction was completed, the reaction mixture was extracted with ethyl acetate three times. Then the filtrates were dried over $Na_2SO_4$ and concentrated under reduced pressure. The residue obtained was purified by chromatography (silica gel, PE–EtOAc, 100:1) to give the product.

## III. Fluorescence spectra of 3a, 3e and 3m.

The experimental results on the fluorescence spectra of **3a, 3e** and **3m** were shown in Figure 1.

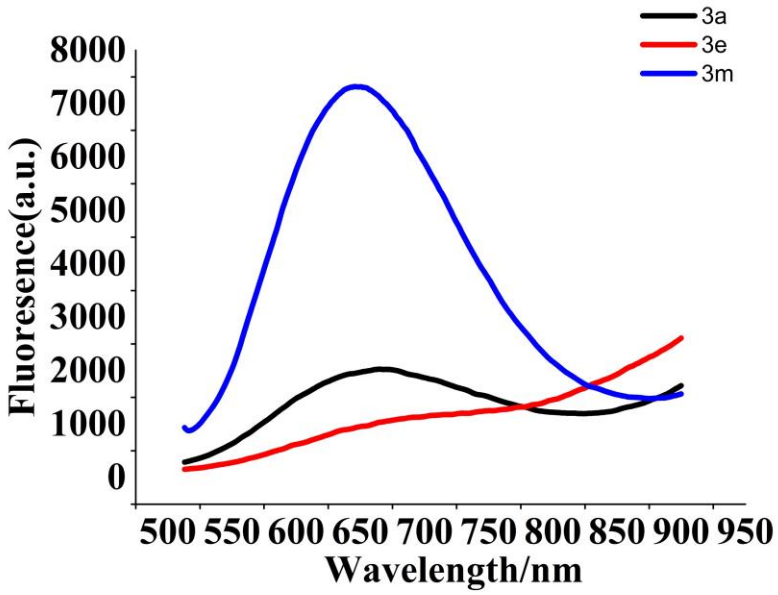

## IV. References

(1) S. Chowdhury, S. Roy, *J. Org. Chem.*, 1997, **62**, 199-200.

(2) C. Kuang, Q.Yang, H. Senboku, M. Tokuda, *Tetrahedron*, 2005, **61**, 4043-4052.

(3) C. Kuang, Q. Yang, H. Senboku, *Tetrahedron Lett.*, 2001, **42**, 3893-3896.

## V. $^{1}$H and $^{13}$C NMR spectra of the synthetic compounds

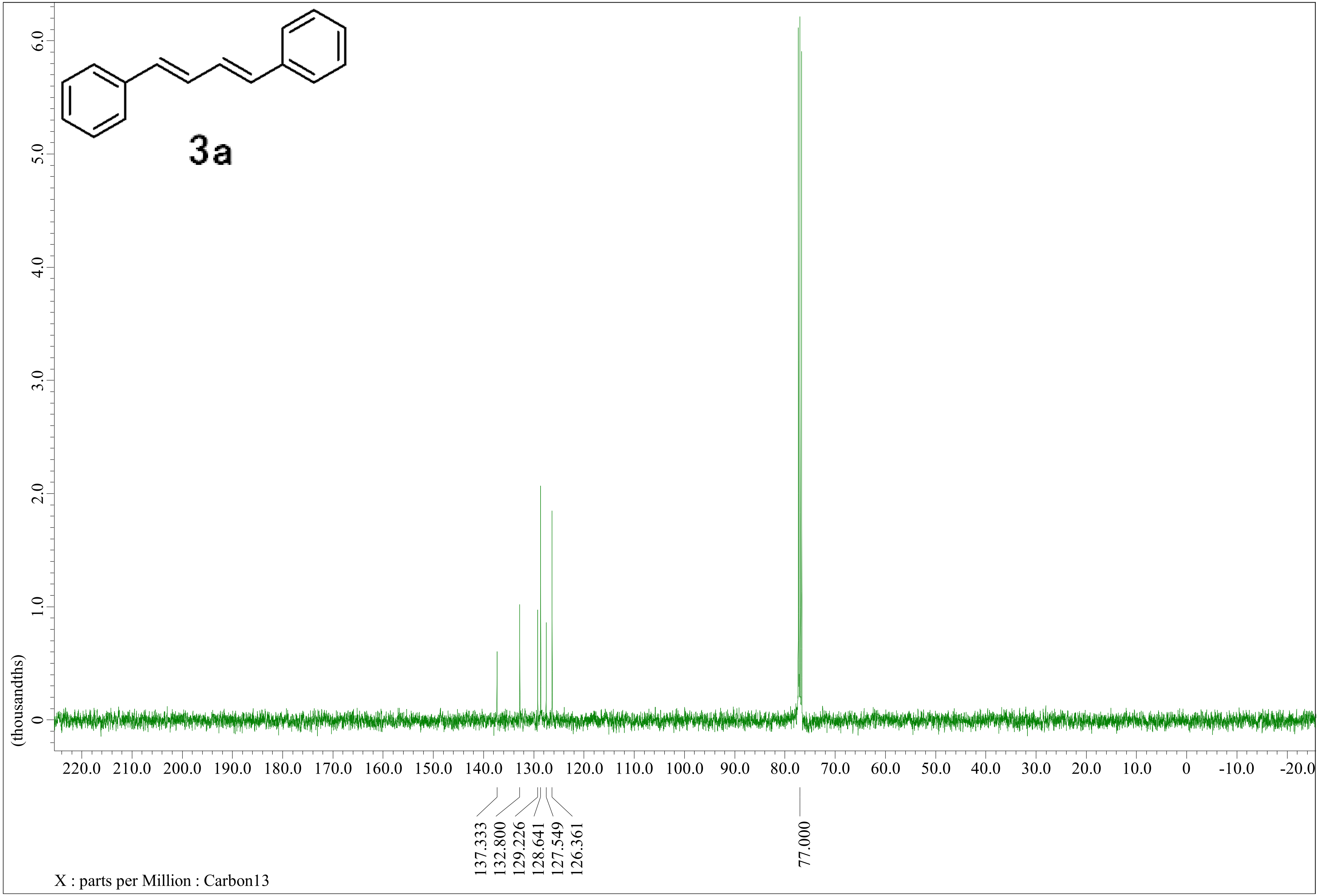
3a
(thousandths)
6.0
5.0
4.0
3.0
2.0
1.0
0
220.0 210.0 200.0 190.0 180.0 170.0 160.0 150.0 140.0 130.0 120.0 110.0 100.0 90.0 80.0 70.0 60.0 50.0 40.0 30.0 20.0 10.0 0 -10.0 -20.0
137.333
132.800
129.226
128.641
127.549
126.361
77.000
X : parts per Million : Carbon13

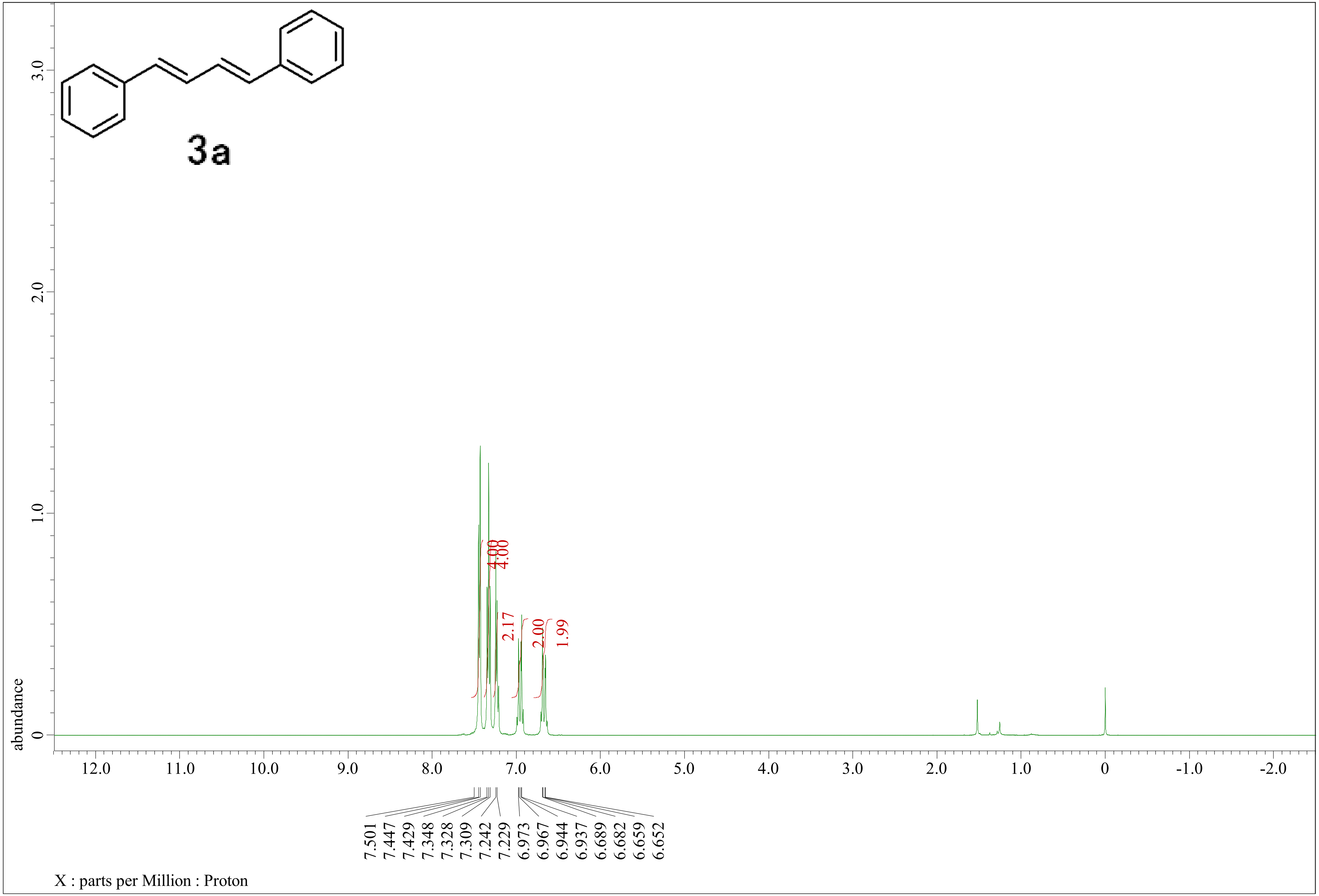
3a
abundance
3.0
2.0
1.0
0
4.00
4.00
2.17
2.00
1.99
12.0 11.0 10.0 9.0 8.0 7.0 6.0 5.0 4.0 3.0 2.0 1.0 0 -1.0 -2.0
7.501 7.447 7.429 7.348 7.328 7.309 7.242 7.229 6.973 6.967 6.944 6.937 6.689 6.682 6.659 6.652
X : parts per Million : Proton

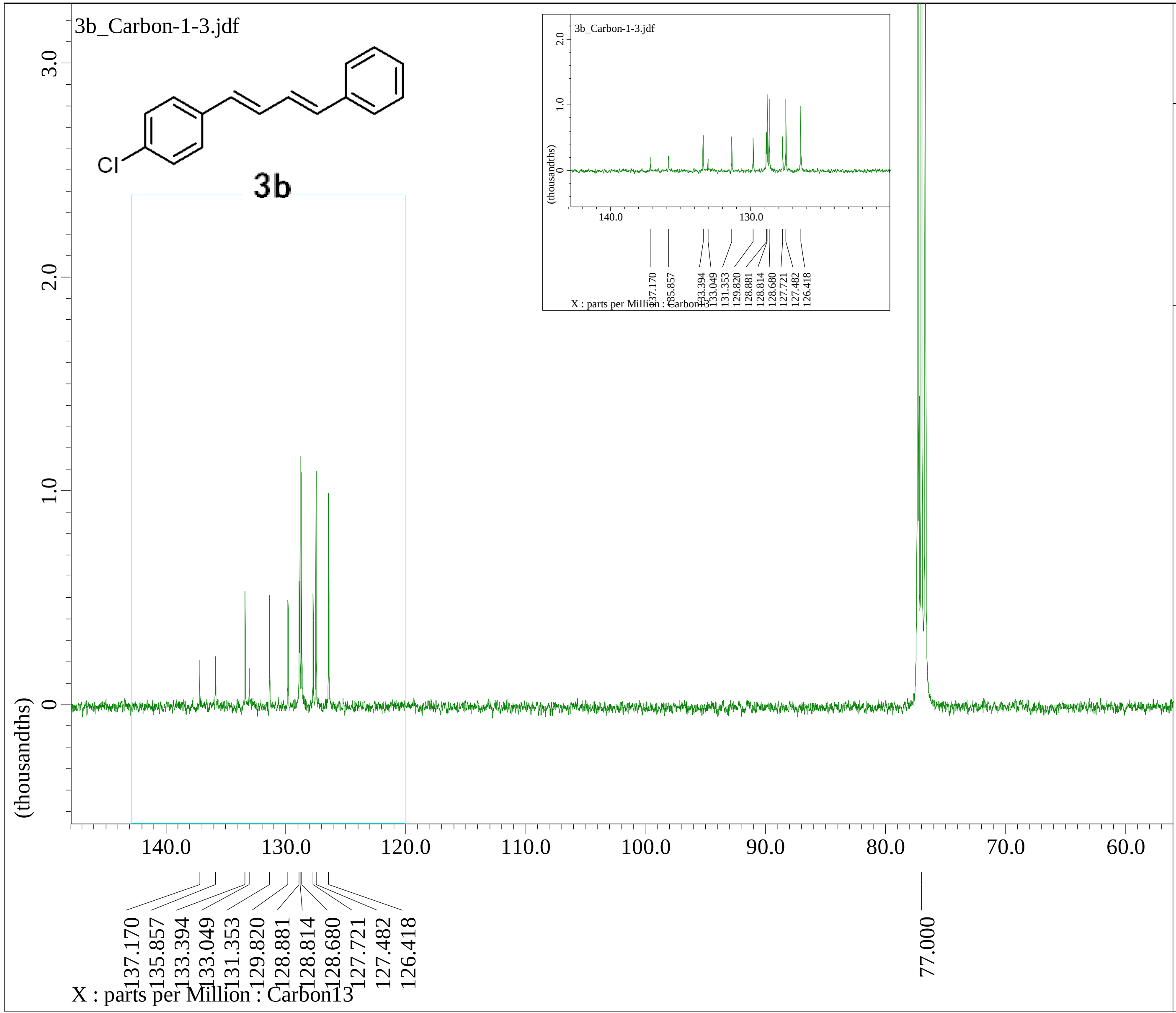


```
---- PROCESSING PARAMETERS ----
sexp( 2.0[Hz], 0.0[s] )
trapezoid( 0[%], 0[%], 80[%], 100[%] )
zerofill( 1 )
fft( 1, TRUE, TRUE )
machinephase
ppm

Derived from: 3b_Carbon-1-1.jdf

Filename            = 3b_Carbon-1-3.j
Author              = delta
Experiment          = carbon.jxp
Sample_Id           = 3b
Solvent             = CHLOROFORM-D
Actual_Start_Time   = 24-JAN-2019 12:
Revision_Time       =  4-FEB-2019 11:

Comment             = single pulse de
Data_Format         = 1D COMPLEX
Dim_Size            = 26214
Dim_Title           = Carbon13
Dim_Units           = [ppm]
Dimensions          = X
Spectrometer        = JNM-ECZ400S/L1

Field_Strength      = 9.389766[T] (40
X_Acq_Duration      = 1.03809024[s]
X_Domain            = 13C
X_Freq              = 100.52530333[MH
X_Offset            = 100[ppm]
X_Points            = 32768
X_Prescans          = 4
X_Resolution        = 0.96330739[Hz]
X_Sweep             = 31.56565657[kHz
X_Sweep_Clipped     = 25.25252525[kHz
Irr_Domain          = Proton
Irr_Freq            = 399.78219838[MH
Irr_Offset          = 5[ppm]
Clipped             = FALSE
Scans               = 4000
Total_Scans         = 4000

Relaxation_Delay    = 2[s]
Recvr_Gain          = 50
Temp_Get            = 21.7[dC]
X_90_Width          = 8.8[us]
X_Acq_Time          = 1.03809024[s]
X_Angle             = 30[deg]
X_Atn               = 3.4[dB]
X_Pulse             = 2.93333333[us]
```

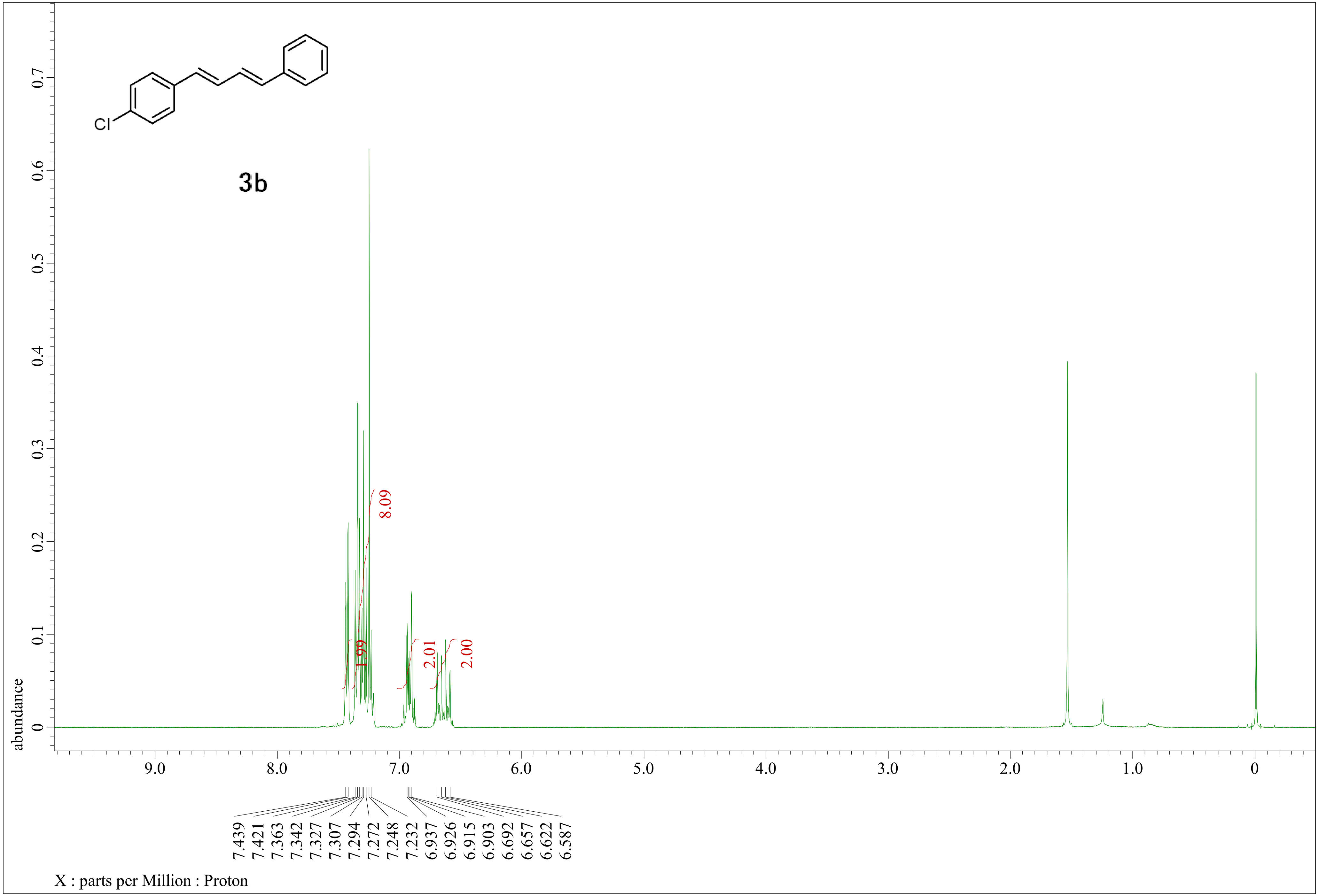

Cl
3b
abundance
0 0.1 0.2 0.3 0.4 0.5 0.6 0.7
8.09
1.99
2.01
2.00
9.0 8.0 7.0 6.0 5.0 4.0 3.0 2.0 1.0 0
7.439 7.421 7.363 7.342 7.327 7.307 7.294 7.272 7.248 7.232 6.937 6.926 6.915 6.903 6.692 6.657 6.622 6.587
X : parts per Million : Proton

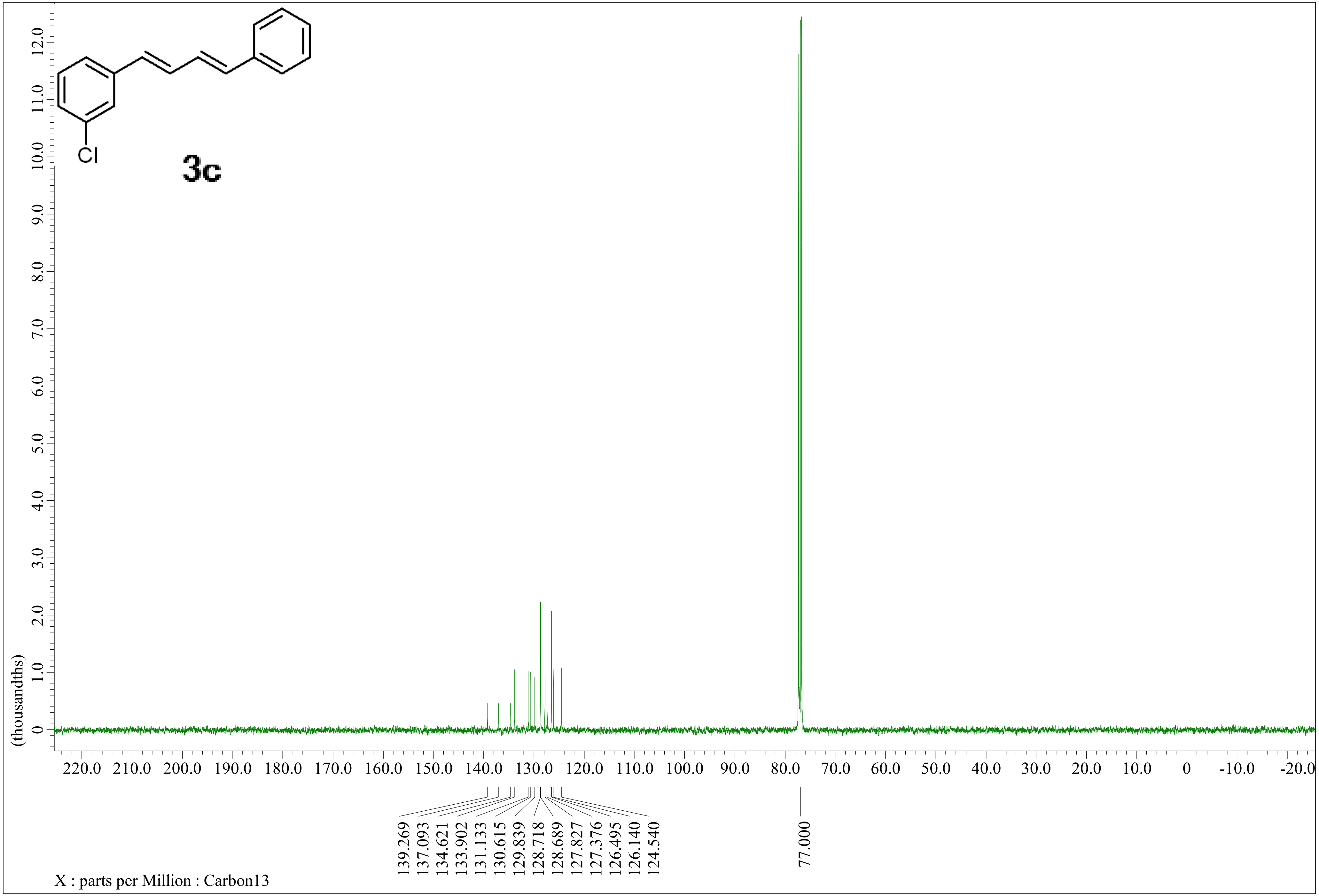

Cl
3c
(thousandths)
12.0
11.0
10.0
9.0
8.0
7.0
6.0
5.0
4.0
3.0
2.0
1.0
0
220.0 210.0 200.0 190.0 180.0 170.0 160.0 150.0 140.0 130.0 120.0 110.0 100.0 90.0 80.0 70.0 60.0 50.0 40.0 30.0 20.0 10.0 0 -10.0 -20.0
139.269
137.093
134.621
133.902
131.133
130.615
129.839
128.718
128.689
127.827
127.376
126.495
126.140
124.540
77.000
X : parts per Million : Carbon13

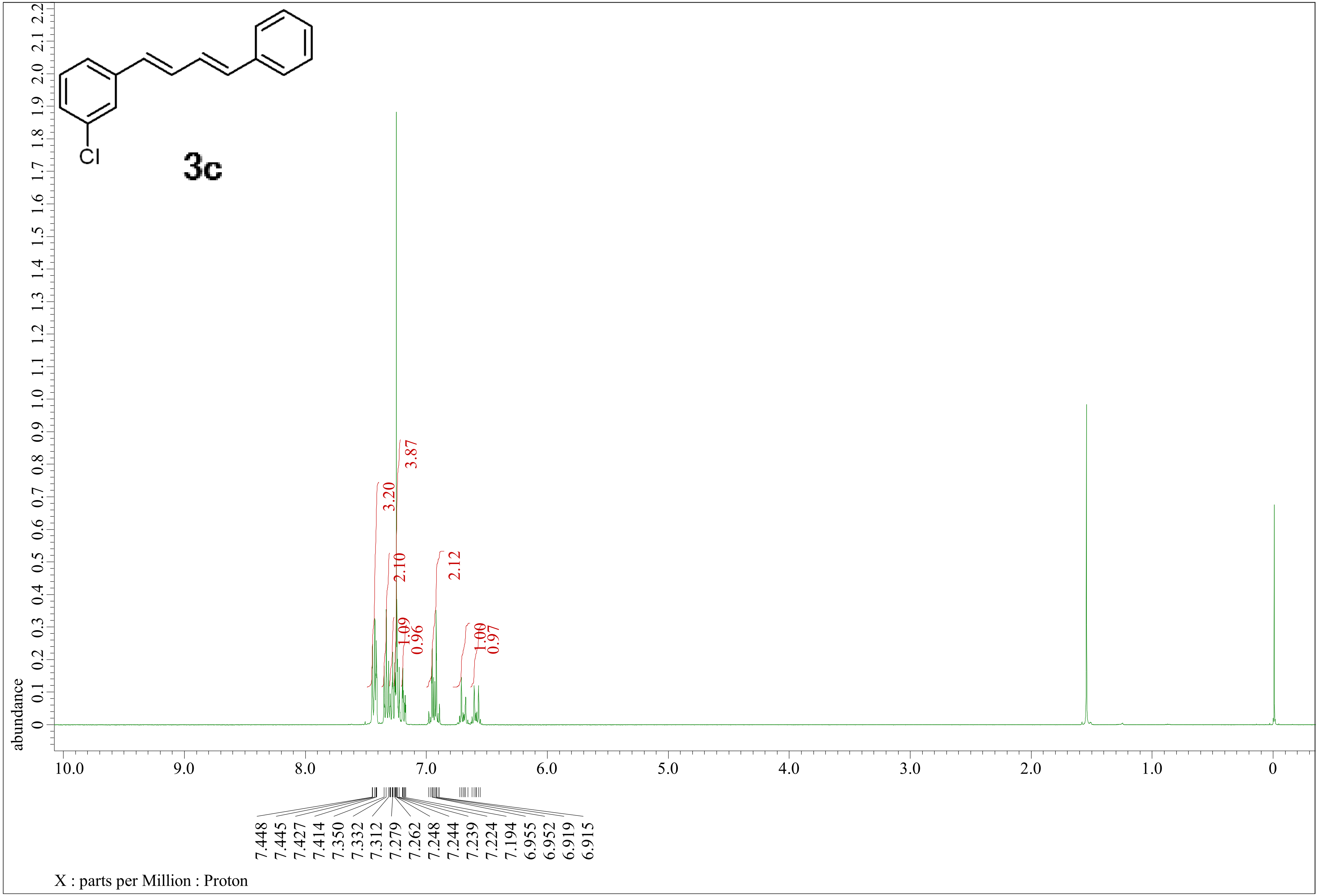

3c
Cl
abundance
X : parts per Million : Proton
3.20
2.10
3.87
1.09
0.96
2.12
1.00
0.97
7.448
7.445
7.427
7.414
7.350
7.332
7.312
7.279
7.262
7.248
7.244
7.239
7.224
7.194
6.955
6.952
6.919
6.915

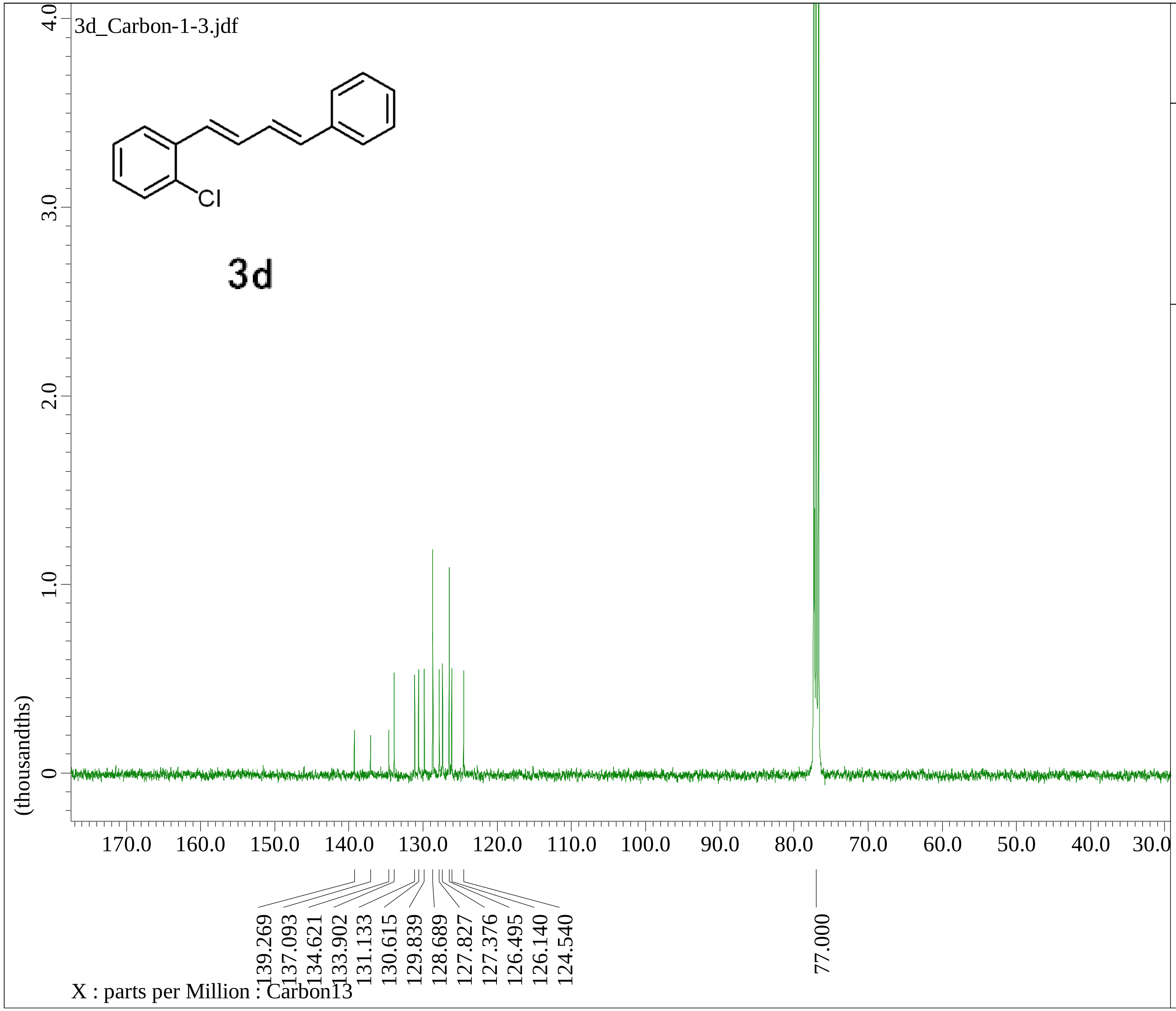


---- PROCESSING PARAMETERS ----
sexp( 2.0[Hz], 0.0[s] )
trapezoid( 0[%], 0[%], 80[%], 100[%] )
zerofill( 1 )
fft( 1, TRUE, TRUE )
machinephase
ppm

Derived from: 3d_Carbon-1-1.jdf

| | |
|---|---|
| Filename | = 3d_Carbon-1-3.j |
| Author | = delta |
| Experiment | = carbon.jxp |
| Sample_Id | = 3d |
| Solvent | = CHLOROFORM-D |
| Actual_Start_Time | = 24-JAN-2019 16: |
| Revision_Time | = 4-FEB-2019 11: |
| Comment | = single pulse de |
| Data_Format | = 1D COMPLEX |
| Dim_Size | = 26214 |
| Dim_Title | = Carbon13 |
| Dim_Units | = [ppm] |
| Dimensions | = X |
| Spectrometer | = JNM-ECZ400S/L1 |
| Field_Strength | = 9.389766[T] (40 |
| X_Acq_Duration | = 1.03809024[s] |
| X_Domain | = 13C |
| X_Freq | = 100.52530333[MH |
| X_Offset | = 100[ppm] |
| X_Points | = 32768 |
| X_Prescans | = 4 |
| X_Resolution | = 0.96330739[Hz] |
| X_Sweep | = 31.56565657[kHz |
| X_Sweep_Clipped | = 25.25252525[kHz |
| Irr_Domain | = Proton |
| Irr_Freq | = 399.78219838[MH |
| Irr_Offset | = 5[ppm] |
| Clipped | = FALSE |
| Scans | = 4000 |
| Total_Scans | = 4000 |
| Relaxation_Delay | = 2[s] |
| Recvr_Gain | = 50 |
| Temp_Get | = 23.6[dC] |
| X_90_Width | = 8.8[us] |
| X_Acq_Time | = 1.03809024[s] |
| X_Angle | = 30[deg] |
| X_Atn | = 3.4[dB] |
| X_Pulse | = 2.93333333[us] |

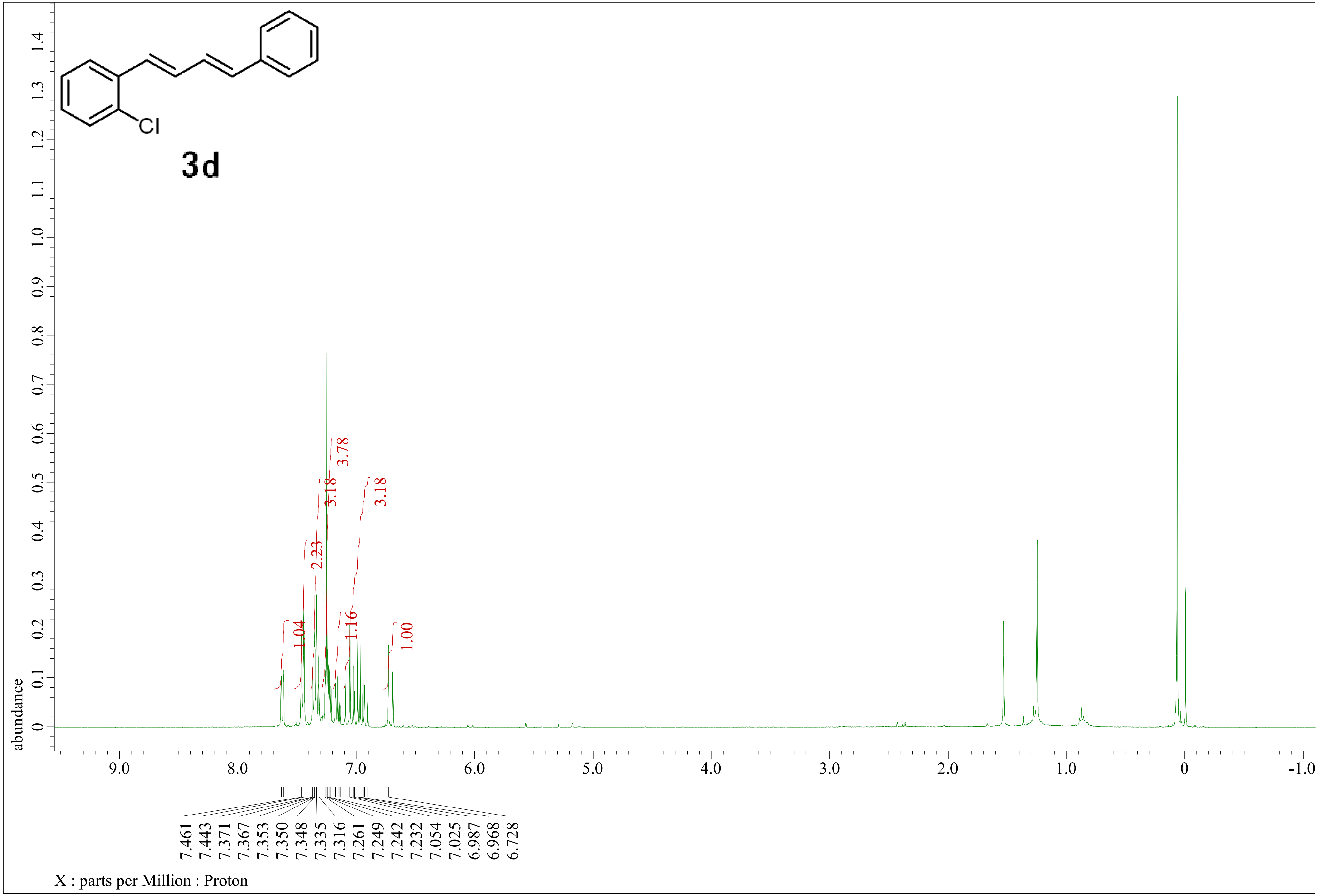

Cl
3d
abundance
1.4
1.3
1.2
1.1
1.0
0.9
0.8
0.7
0.6
0.5
0.4
0.3
0.2
0.1
0
1.04
2.23
3.18
3.78
1.16
3.18
1.00
9.0
8.0
7.0
6.0
5.0
4.0
3.0
2.0
1.0
0
-1.0
7.461
7.443
7.371
7.367
7.353
7.350
7.348
7.335
7.316
7.261
7.249
7.242
7.232
7.054
7.025
6.987
6.968
6.728
X : parts per Million : Proton

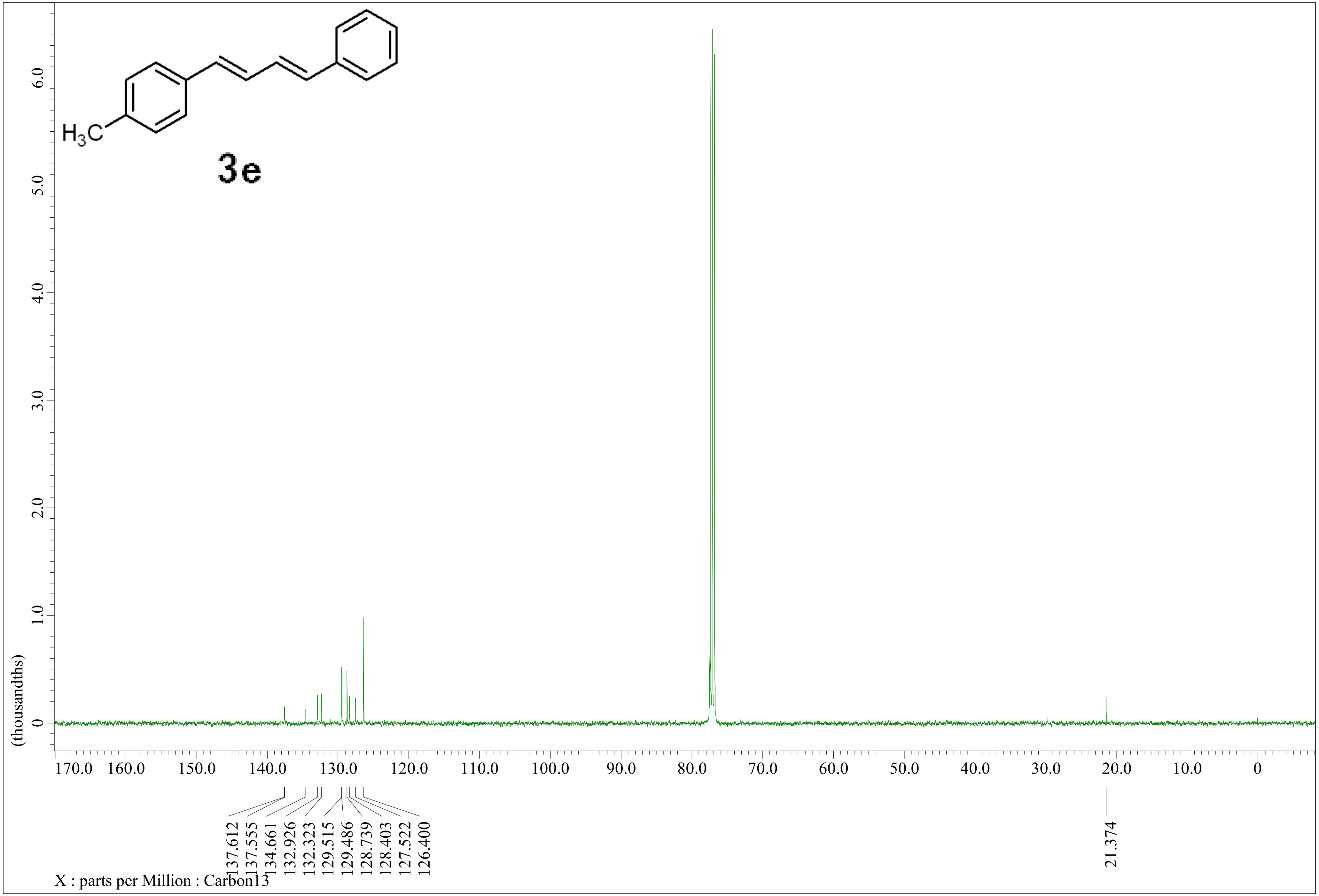

H3C
3e
6.0
5.0
4.0
3.0
2.0
1.0
0
(thousandths)
170.0 160.0 150.0 140.0 130.0 120.0 110.0 100.0 90.0 80.0 70.0 60.0 50.0 40.0 30.0 20.0 10.0 0
137.612
137.555
134.661
132.926
132.323
129.515
129.486
128.739
128.403
127.522
126.400
21.374
X : parts per Million : Carbon13

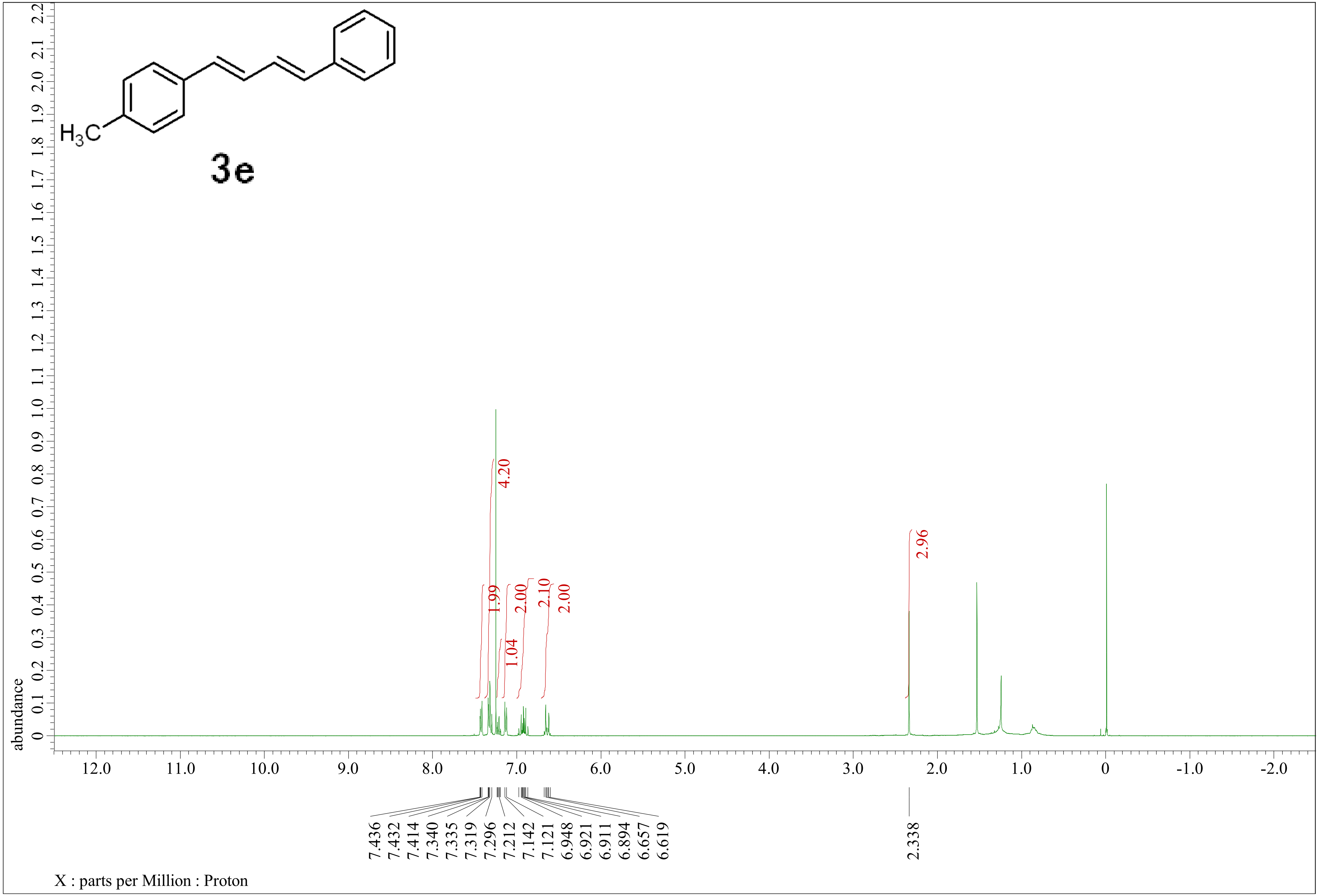

3e
H3C
abundance
0 0.1 0.2 0.3 0.4 0.5 0.6 0.7 0.8 0.9 1.0 1.1 1.2 1.3 1.4 1.5 1.6 1.7 1.8 1.9 2.0 2.1 2.2
12.0 11.0 10.0 9.0 8.0 7.0 6.0 5.0 4.0 3.0 2.0 1.0 0 -1.0 -2.0
4.20
1.99
1.04
2.00
2.10
2.00
2.96
7.436 7.432 7.414 7.340 7.335 7.319 7.296 7.212 7.142 7.121 6.948 6.921 6.911 6.894 6.657 6.619
2.338
X : parts per Million : Proton

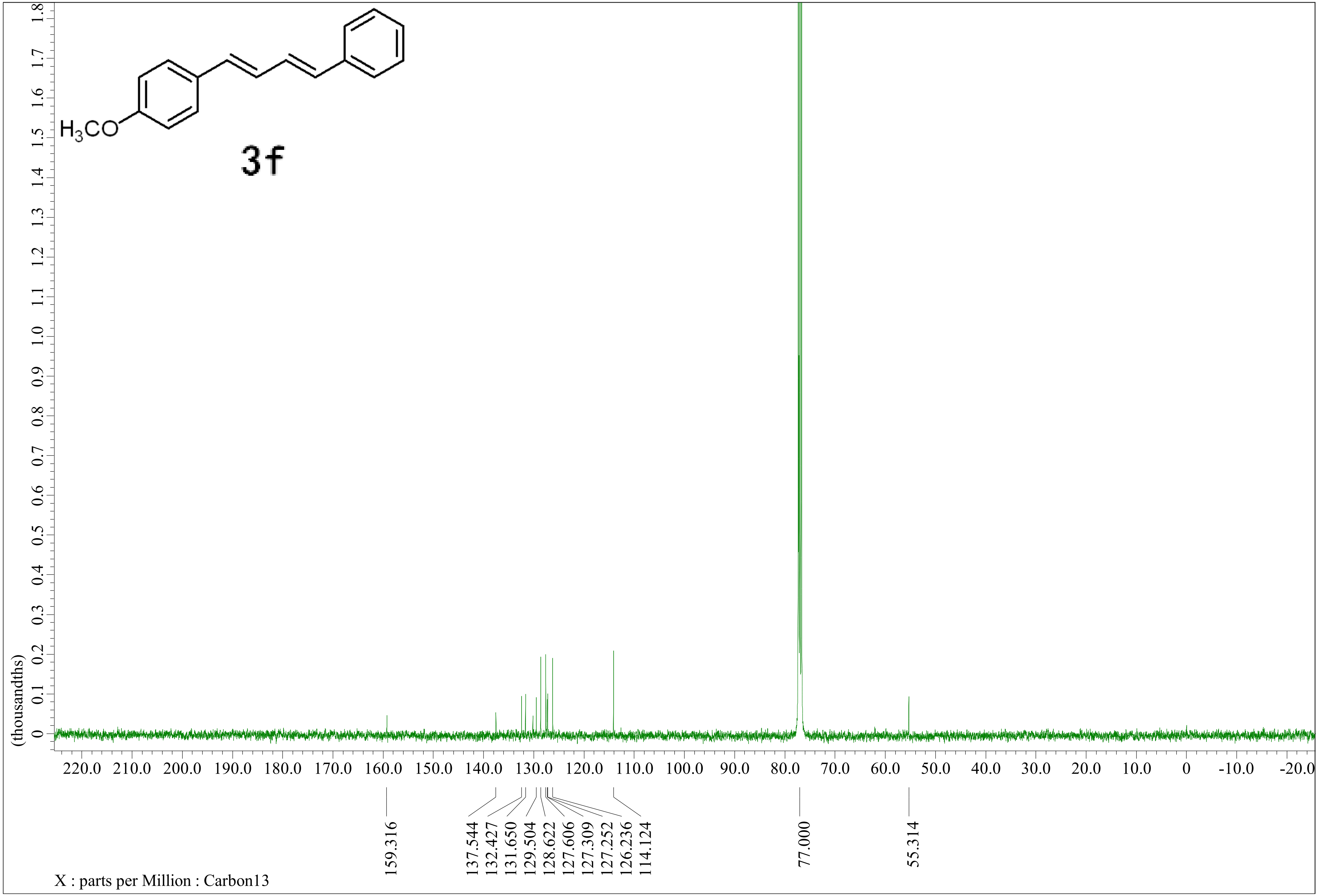
H3CO
3f
(thousandths)
0
0.1
0.2
0.3
0.4
0.5
0.6
0.7
0.8
0.9
1.0
1.1
1.2
1.3
1.4
1.5
1.6
1.7
1.8
220.0 210.0 200.0 190.0 180.0 170.0 160.0 150.0 140.0 130.0 120.0 110.0 100.0 90.0 80.0 70.0 60.0 50.0 40.0 30.0 20.0 10.0 0 -10.0 -20.0
159.316
137.544
132.427
131.650
129.504
128.622
127.606
127.309
127.252
126.236
114.124
77.000
55.314
X : parts per Million : Carbon13

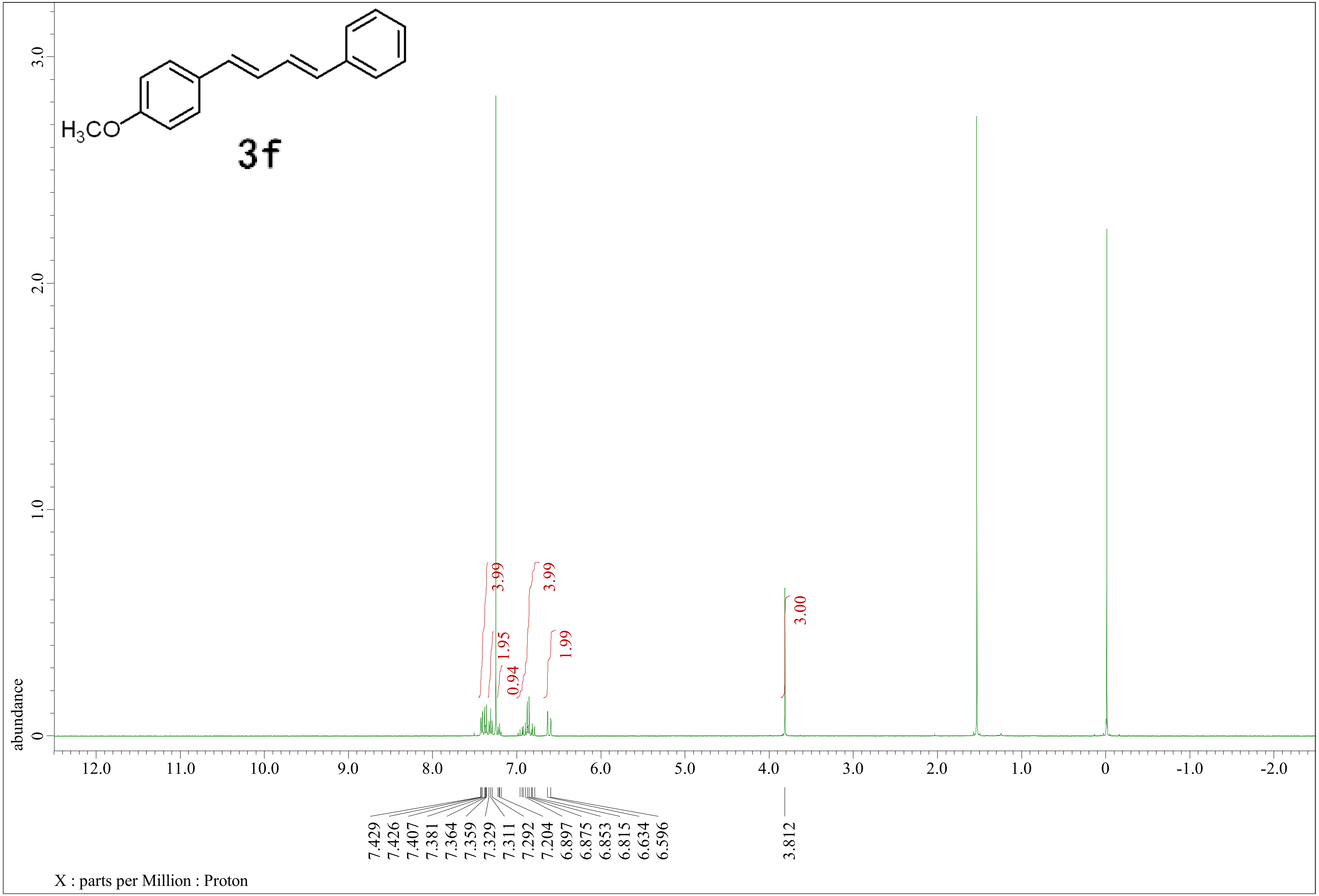
H3CO
3f
abundance
3.0
2.0
1.0
0
3.99
1.95
0.94
3.99
1.99
3.00
12.0
11.0
10.0
9.0
8.0
7.0
6.0
5.0
4.0
3.0
2.0
1.0
0
-1.0
-2.0
7.429
7.426
7.407
7.381
7.364
7.359
7.329
7.311
7.292
7.204
6.897
6.875
6.853
6.815
6.634
6.596
3.812
X : parts per Million : Proton

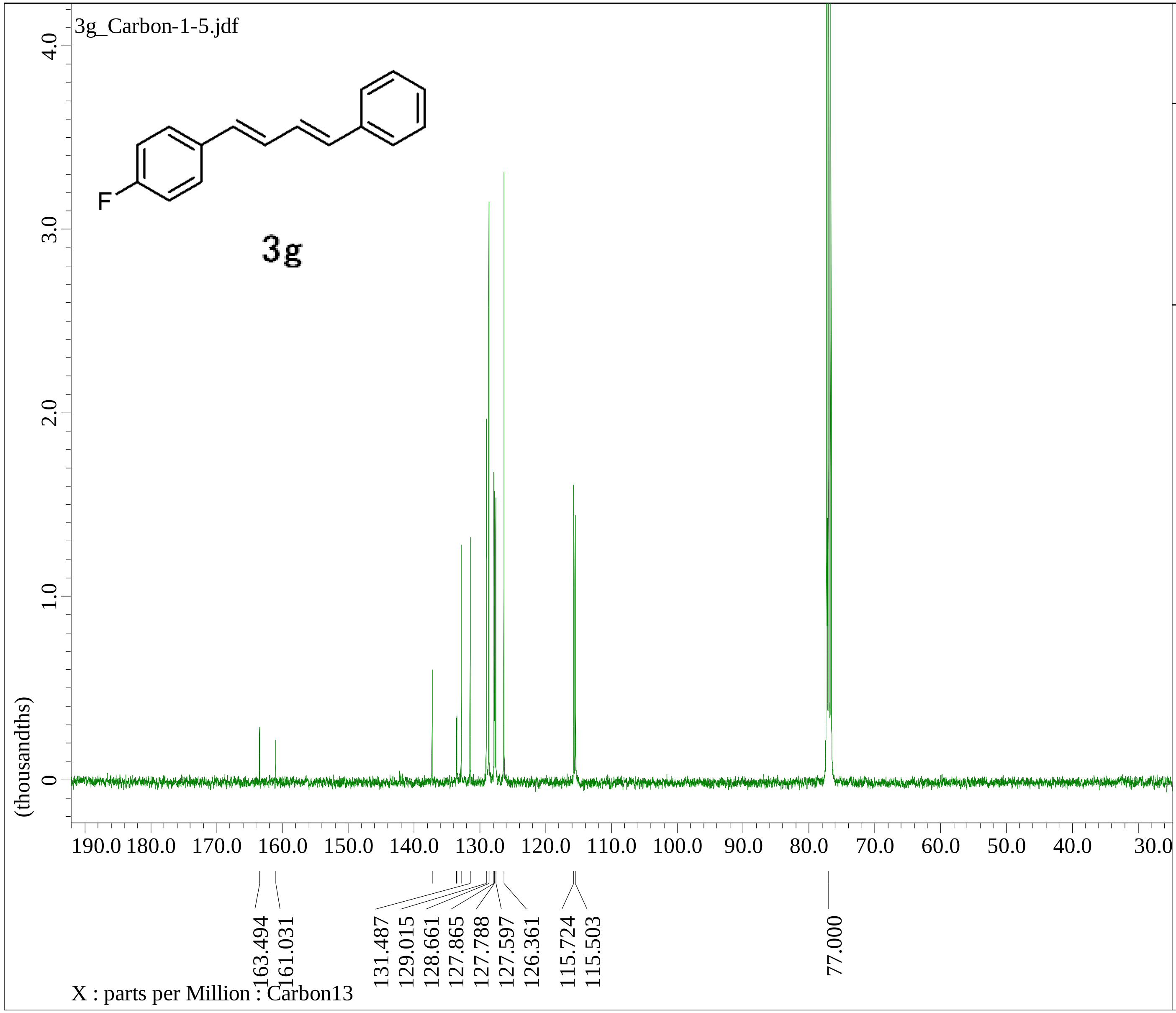


JEOL

```
---- PROCESSING PARAMETERS ----
sexp( 2.0[Hz], 0.0[s] )
trapezoid( 0[%], 0[%], 80[%], 100[%] )
zerofill( 1 )
fft( 1, TRUE, TRUE )
machinephase
ppm

Derived from: 3g_Carbon-1-1.jdf
```

```
Filename              = 3g_Carbon-1-5.j
Author                = delta
Experiment            = carbon.jxp
Sample_Id             = 3g
Solvent               = CHLOROFORM-D
Actual_Start_Time     = 24-JAN-2019 19:
Revision_Time         =  4-FEB-2019 11:

Comment               = single pulse de
Data_Format           = 1D COMPLEX
Dim_Size              = 26214
Dim_Title             = Carbon13
Dim_Units             = [ppm]
Dimensions            = X
Spectrometer          = JNM-ECZ400S/L1

Field_Strength        = 9.389766[T] (40
X_Acq_Duration        = 1.03809024[s]
X_Domain              = 13C
X_Freq                = 100.52530333[MH
X_Offset              = 100[ppm]
X_Points              = 32768
X_Prescans            = 4
X_Resolution          = 0.96330739[Hz]
X_Sweep               = 31.56565657[kHz
X_Sweep_Clipped       = 25.25252525[kHz
Irr_Domain            = Proton
Irr_Freq              = 399.78219838[MH
Irr_Offset            = 5[ppm]
Clipped               = FALSE
Scans                 = 4000
Total_Scans           = 4000

Relaxation_Delay      = 2[s]
Recvr_Gain            = 50
Temp_Get              = 21[dC]
X_90_Width            = 8.8[us]
X_Acq_Time            = 1.03809024[s]
X_Angle               = 30[deg]
X_Atn                 = 3.4[dB]
X_Pulse               = 2.93333333[us]
```

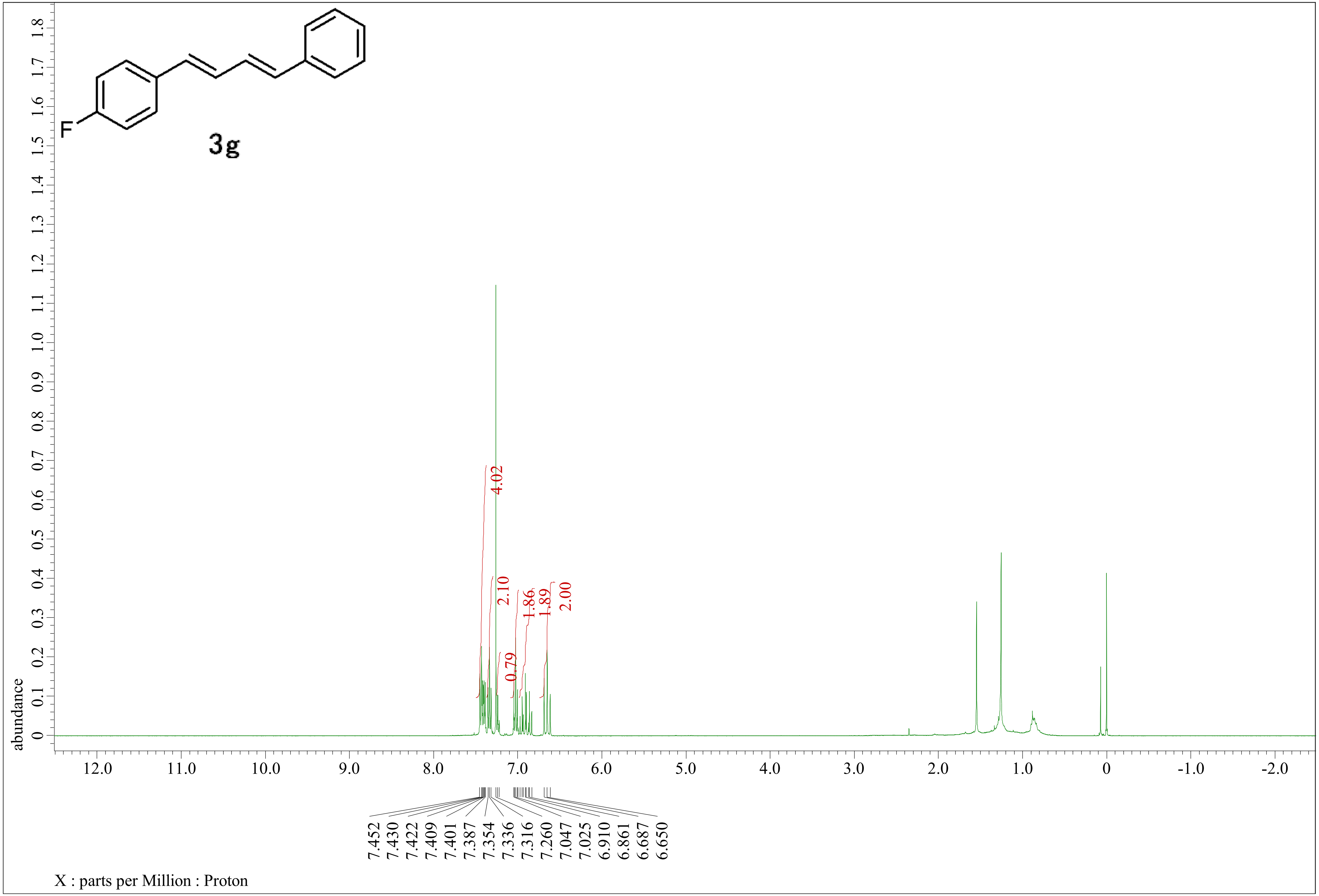
F
3g
abundance
0
0.1
0.2
0.3
0.4
0.5
0.6
0.7
0.8
0.9
1.0
1.1
1.2
1.3
1.4
1.5
1.6
1.7
1.8
4.02
2.10
0.79
1.86
1.89
2.00
12.0
11.0
10.0
9.0
8.0
7.0
6.0
5.0
4.0
3.0
2.0
1.0
0
-1.0
-2.0
7.452
7.430
7.422
7.409
7.401
7.387
7.354
7.336
7.316
7.260
7.047
7.025
6.910
6.861
6.687
6.650
X : parts per Million : Proton

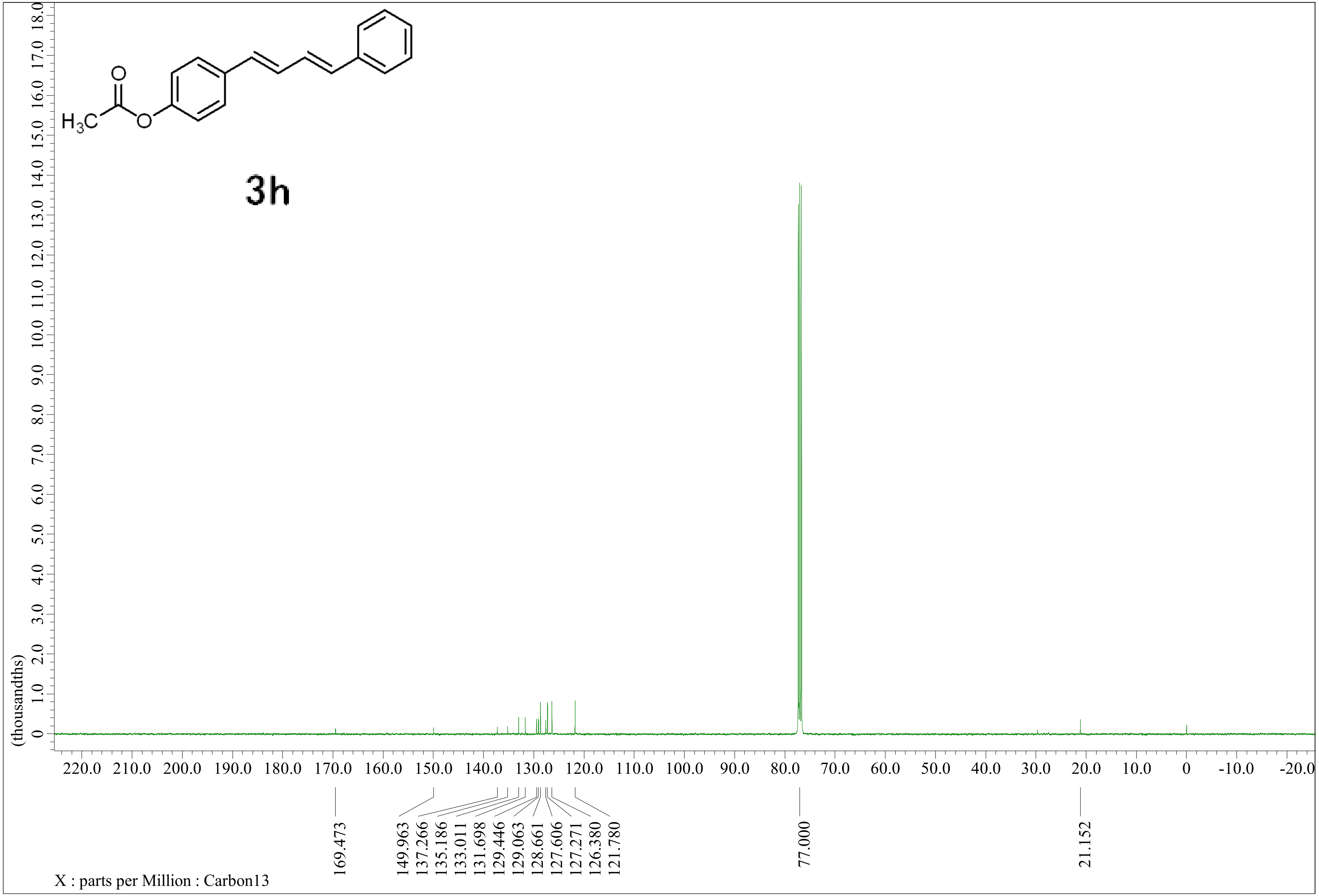

3h
(thousandths)
X : parts per Million : Carbon13
169.473
149.963
137.266
135.186
133.011
131.698
129.446
129.063
128.661
127.606
127.271
126.380
121.780
77.000
21.152

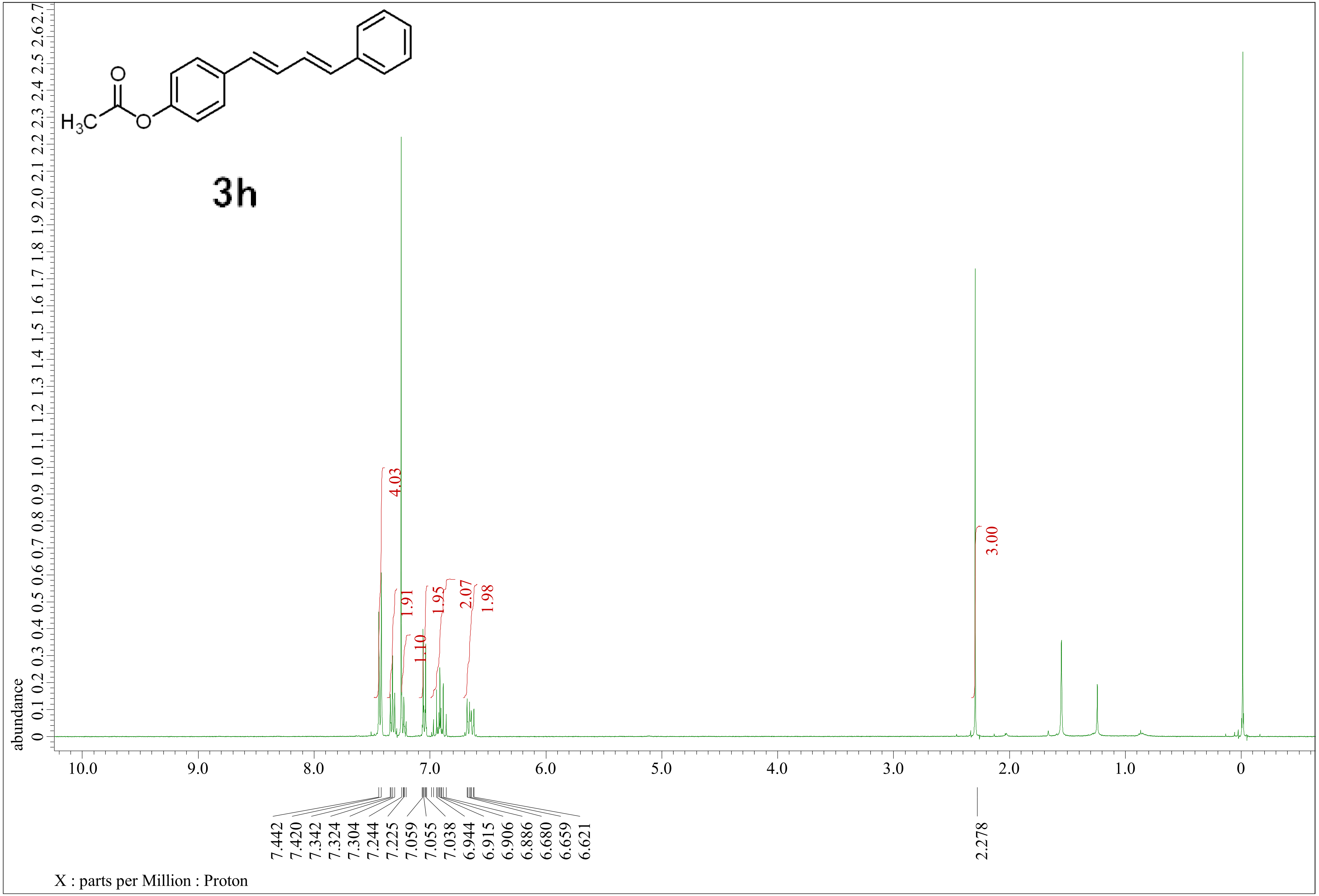

3h
abundance
X : parts per Million : Proton
7.442
7.420
7.342
7.324
7.304
7.244
7.225
7.059
7.055
7.038
6.944
6.915
6.906
6.886
6.680
6.659
6.621
2.278
4.03
1.91
1.10
1.95
2.07
1.98
3.00

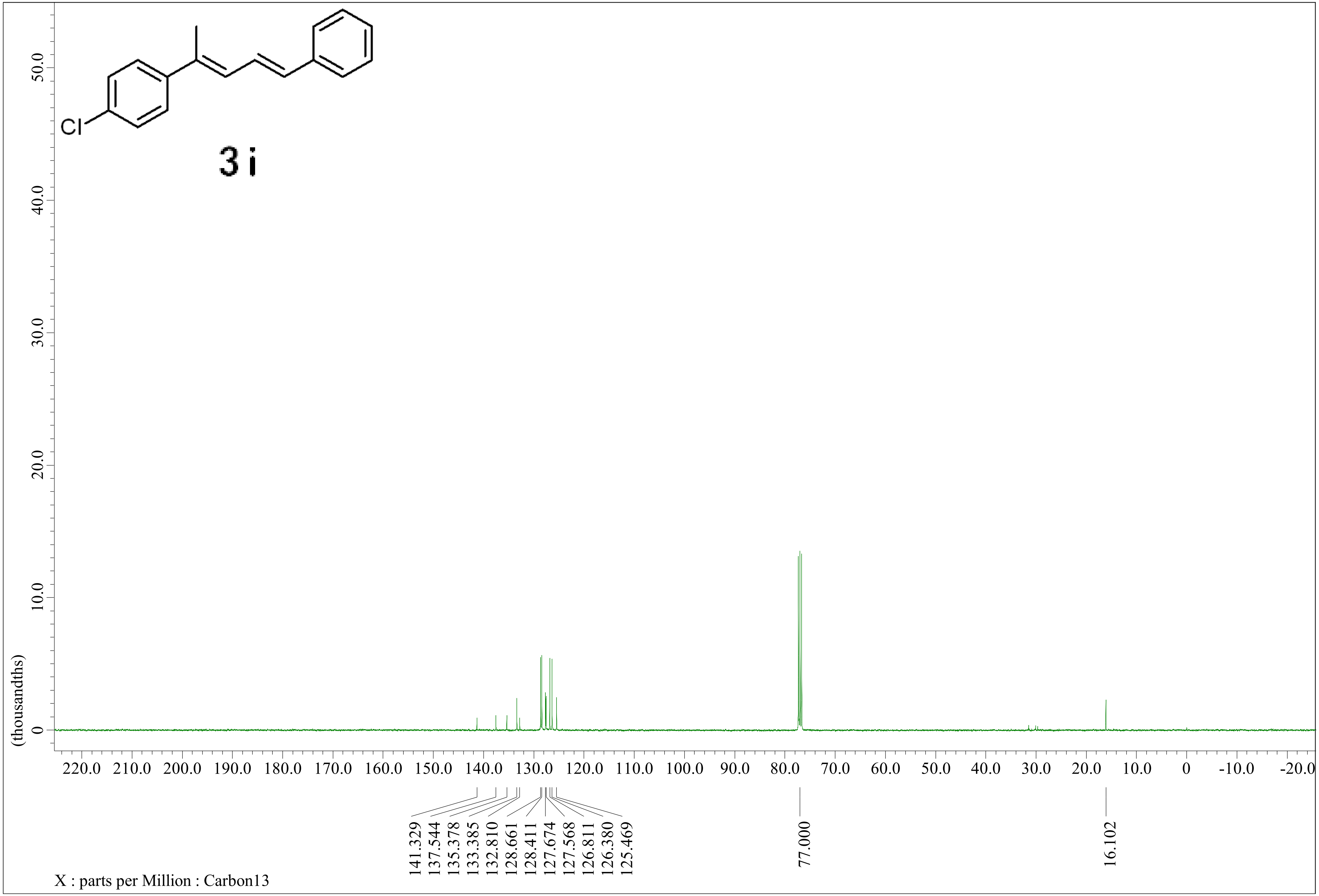

Cl
3i
50.0
40.0
30.0
20.0
10.0
0
(thousandths)
220.0 210.0 200.0 190.0 180.0 170.0 160.0 150.0 140.0 130.0 120.0 110.0 100.0 90.0 80.0 70.0 60.0 50.0 40.0 30.0 20.0 10.0 0 -10.0 -20.0
141.329
137.544
135.378
133.385
132.810
128.661
128.411
127.674
127.568
126.811
126.380
125.469
77.000
16.102
X : parts per Million : Carbon13

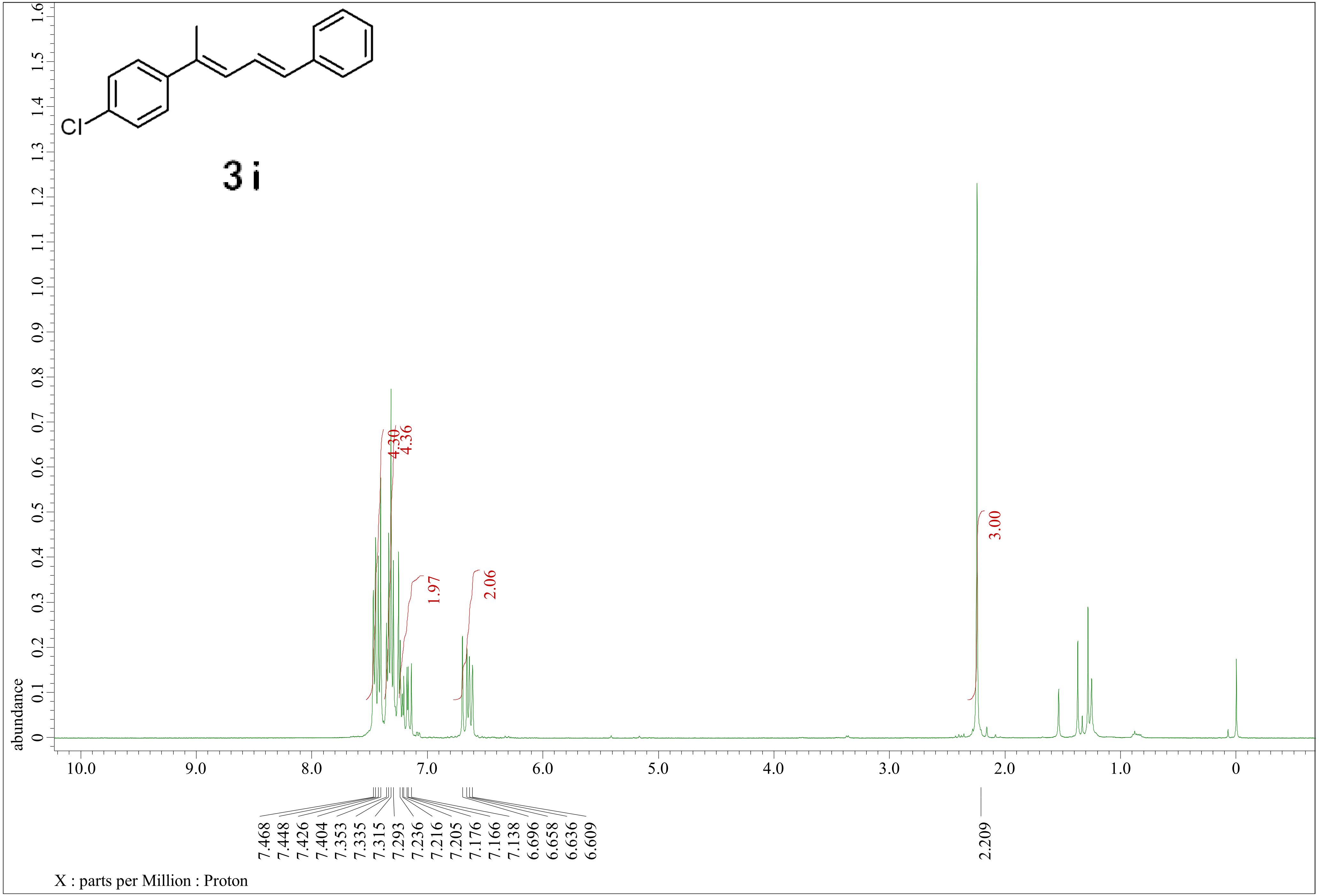

Cl
3i
abundance
1.6
1.5
1.4
1.3
1.2
1.1
1.0
0.9
0.8
0.7
0.6
0.5
0.4
0.3
0.2
0.1
0
4.30
4.36
1.97
2.06
3.00
10.0
9.0
8.0
7.0
6.0
5.0
4.0
3.0
2.0
1.0
0
7.468
7.448
7.426
7.404
7.353
7.335
7.315
7.293
7.236
7.216
7.205
7.176
7.166
7.138
6.696
6.658
6.636
6.609
2.209
X : parts per Million : Proton

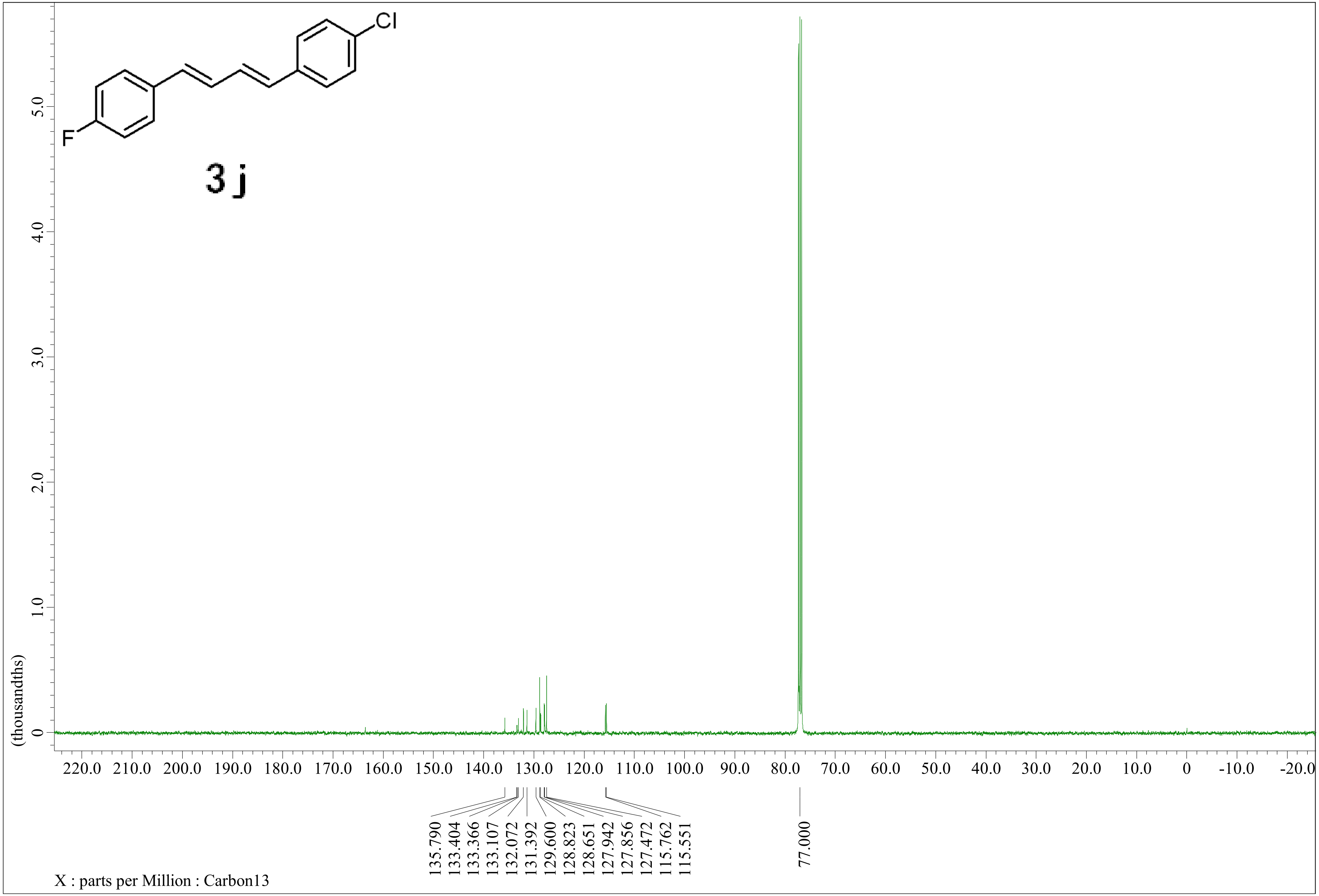
F
Cl
3 j
(thousandths)
5.0
4.0
3.0
2.0
1.0
0
220.0 210.0 200.0 190.0 180.0 170.0 160.0 150.0 140.0 130.0 120.0 110.0 100.0 90.0 80.0 70.0 60.0 50.0 40.0 30.0 20.0 10.0 0 -10.0 -20.0
135.790
133.404
133.366
133.107
132.072
131.392
129.600
128.823
128.651
127.942
127.856
127.472
115.762
115.551
77.000
X : parts per Million : Carbon13

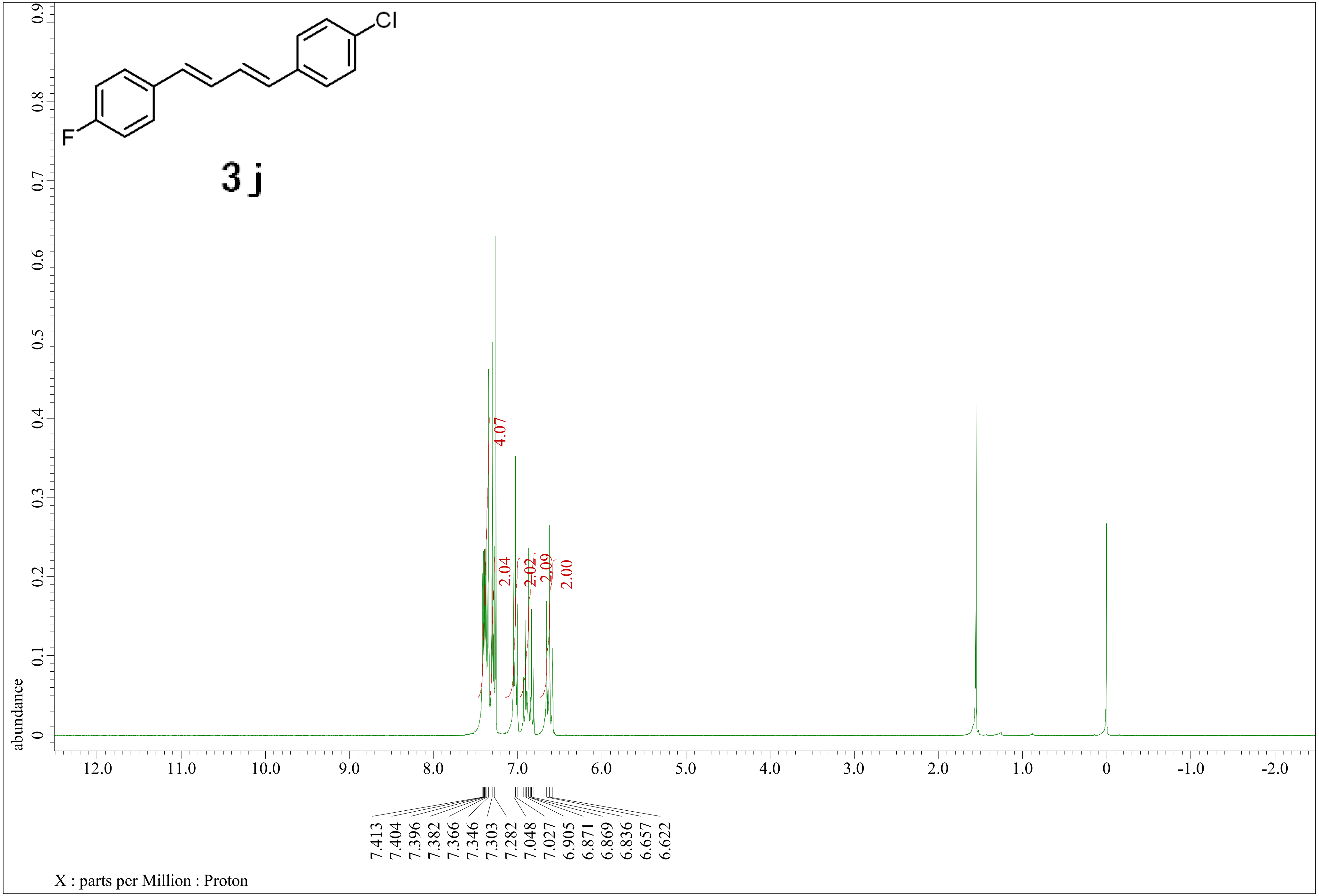

Cl
F
3 j
abundance
0
0.1
0.2
0.3
0.4
0.5
0.6
0.7
0.8
0.9
4.07
2.04
2.02
2.09
2.00
12.0
11.0
10.0
9.0
8.0
7.0
6.0
5.0
4.0
3.0
2.0
1.0
0
-1.0
-2.0
7.413
7.404
7.396
7.382
7.366
7.346
7.303
7.282
7.048
7.027
6.905
6.871
6.869
6.836
6.657
6.622
X : parts per Million : Proton

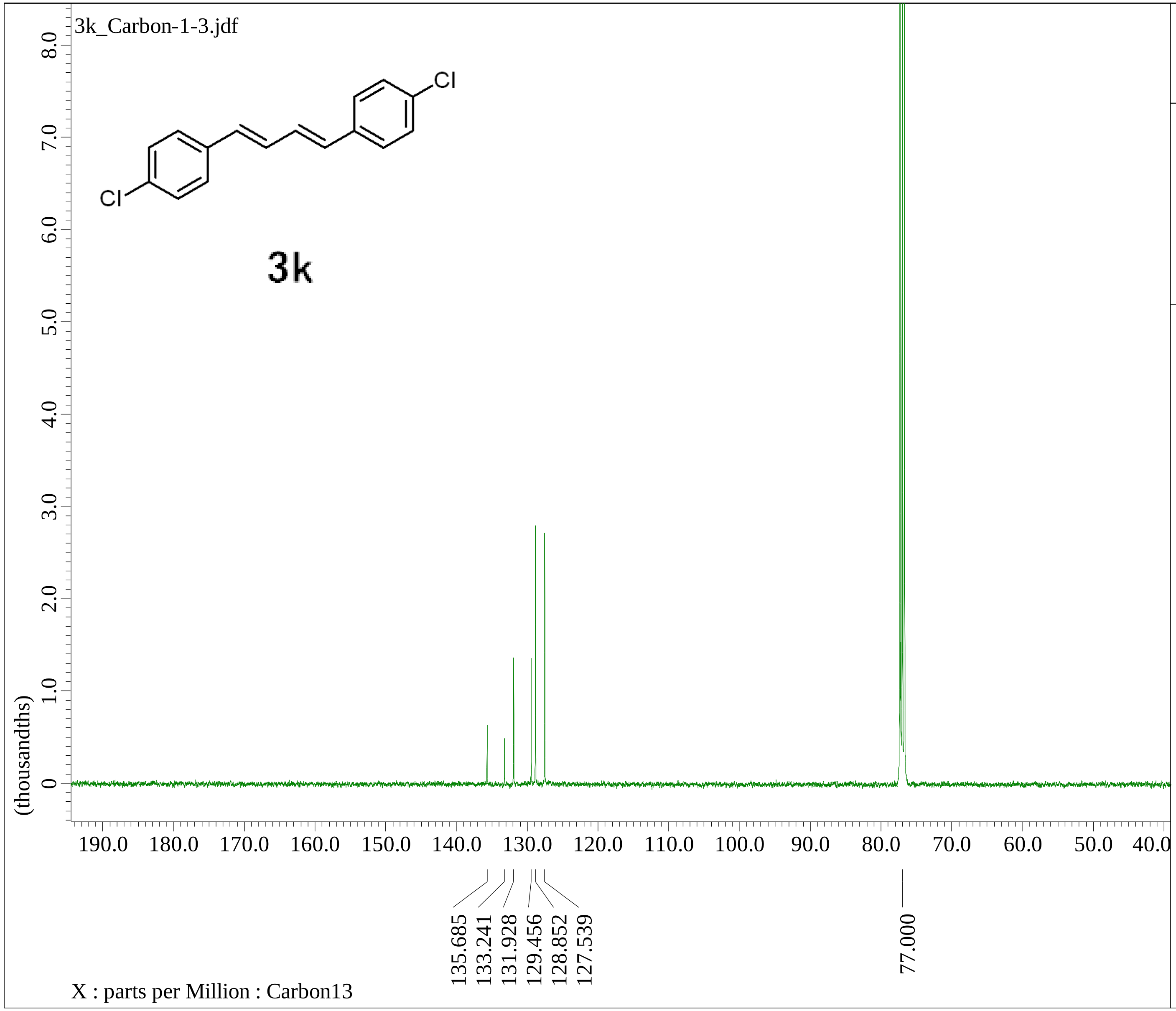


```
---- PROCESSING PARAMETERS ----
sexp( 2.0[Hz], 0.0[s] )
trapezoid( 0[%], 0[%], 80[%], 100[%] )
zerofill( 1 )
fft( 1, TRUE, TRUE )
machinephase
ppm

Derived from: 3k_Carbon-1-1.jdf
```

```
Filename            = 3k_Carbon-1-3.j
Author              = delta
Experiment          = carbon.jxp
Sample_Id           = 3k
Solvent             = CHLOROFORM-D
Actual_Start_Time   = 24-JAN-2019 23:
Revision_Time       =  4-FEB-2019 11:

Comment             = single pulse de
Data_Format         = 1D COMPLEX
Dim_Size            = 26214
Dim_Title           = Carbon13
Dim_Units           = [ppm]
Dimensions          = X
Spectrometer        = JNM-ECZ400S/L1

Field_Strength      = 9.389766[T] (40
X_Acq_Duration      = 1.03809024[s]
X_Domain            = 13C
X_Freq              = 100.52530333[MH
X_Offset            = 100[ppm]
X_Points            = 32768
X_Prescans          = 4
X_Resolution        = 0.96330739[Hz]
X_Sweep             = 31.56565657[kHz
X_Sweep_Clipped     = 25.25252525[kHz
Irr_Domain          = Proton
Irr_Freq            = 399.78219838[MH
Irr_Offset          = 5[ppm]
Clipped             = FALSE
Scans               = 4000
Total_Scans         = 4000

Relaxation_Delay    = 2[s]
Recvr_Gain          = 50
Temp_Get            = 20.6[dC]
X_90_Width          = 8.8[us]
X_Acq_Time          = 1.03809024[s]
X_Angle             = 30[deg]
X_Atn               = 3.4[dB]
X_Pulse             = 2.93333333[us]
```

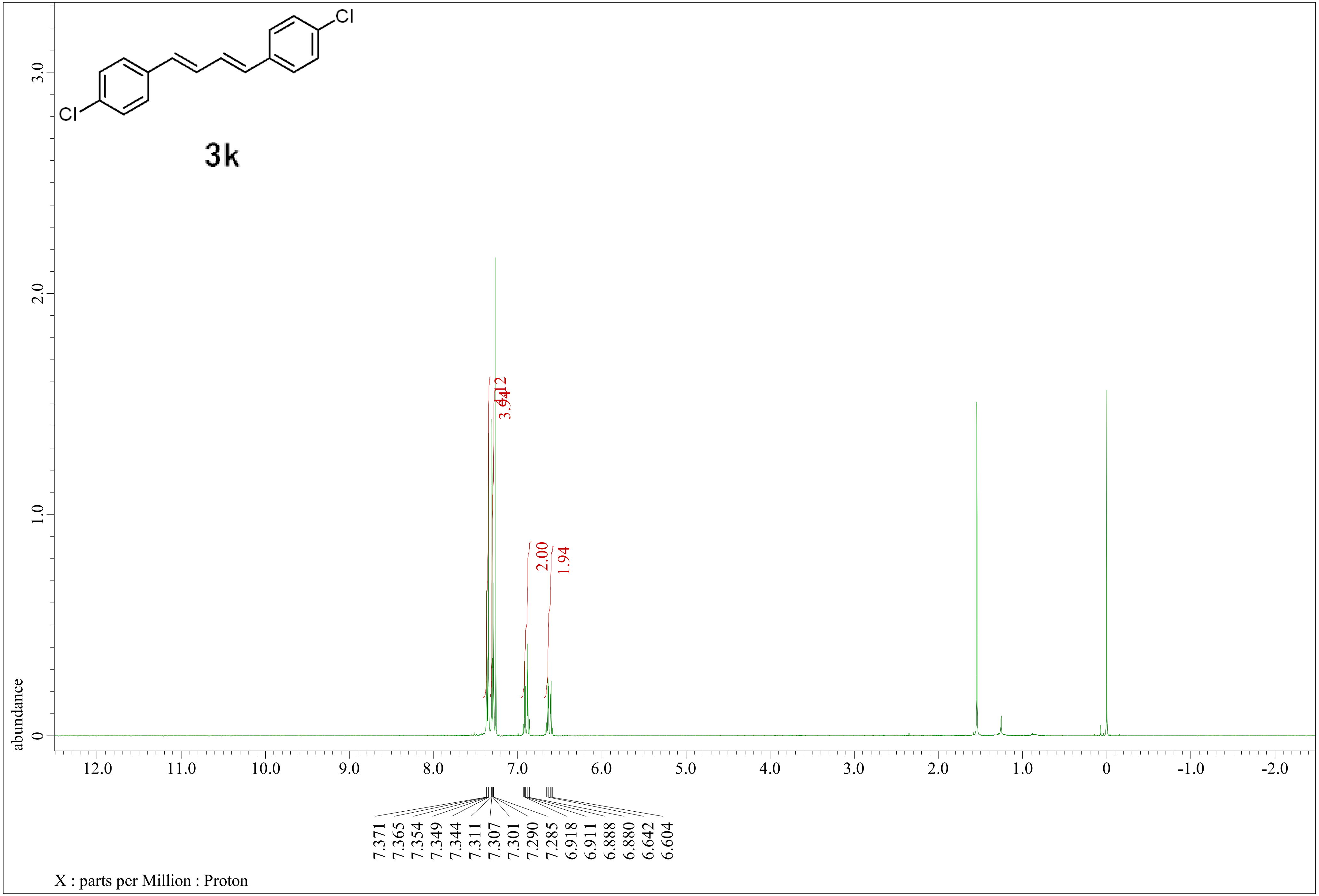
Cl
Cl
3k
3.0
2.0
1.0
0
abundance
4.12
3.94
2.00
1.94
12.0
11.0
10.0
9.0
8.0
7.0
6.0
5.0
4.0
3.0
2.0
1.0
0
-1.0
-2.0
7.371
7.365
7.354
7.349
7.344
7.311
7.307
7.301
7.290
7.285
6.918
6.911
6.888
6.880
6.642
6.604
X : parts per Million : Proton

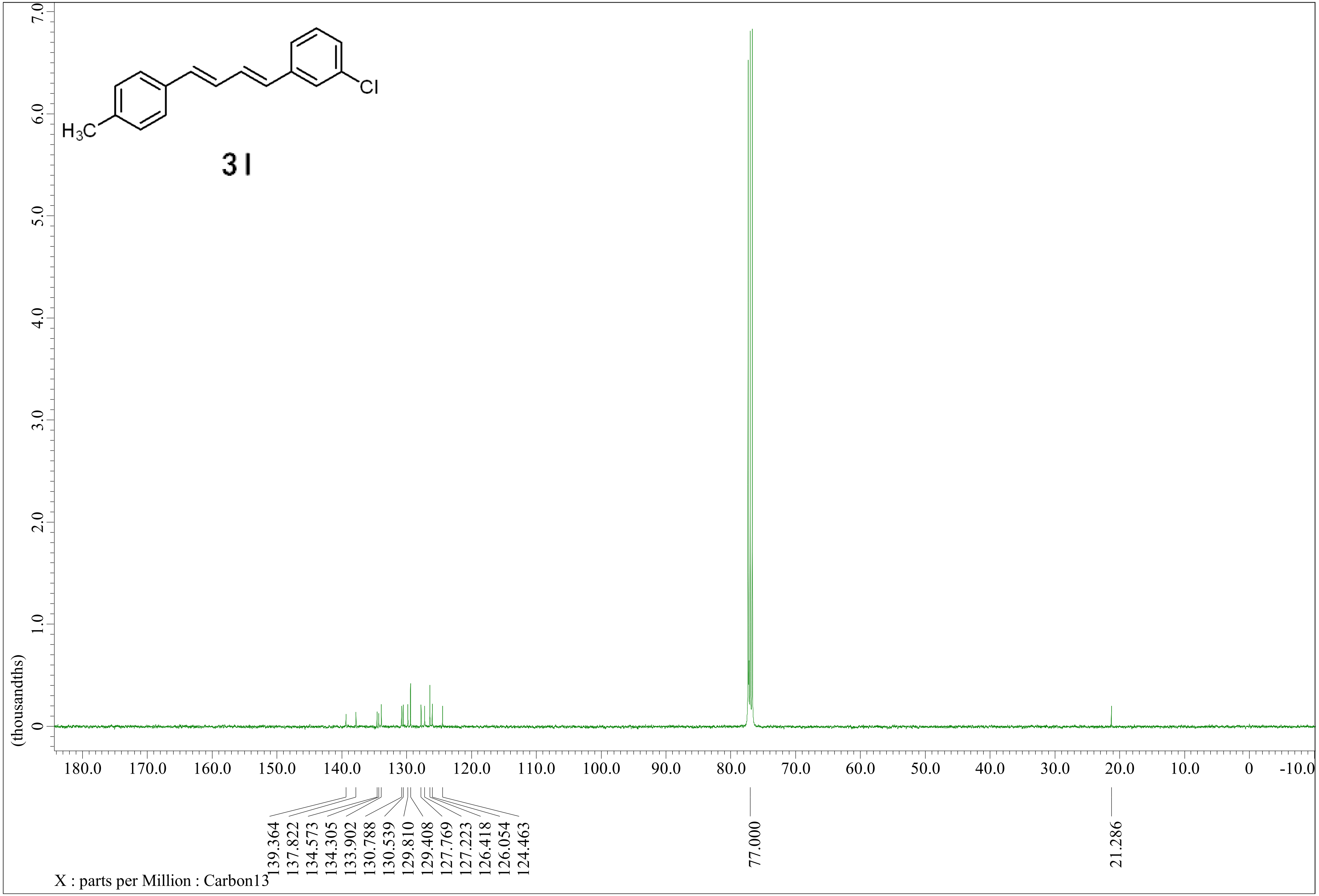

3l
7.0
6.0
5.0
4.0
3.0
2.0
1.0
0
(thousandths)
180.0 170.0 160.0 150.0 140.0 130.0 120.0 110.0 100.0 90.0 80.0 70.0 60.0 50.0 40.0 30.0 20.0 10.0 0 -10.0
139.364
137.822
134.573
134.305
133.902
130.788
130.539
129.810
129.408
127.769
127.223
126.418
126.054
124.463
77.000
21.286
X : parts per Million : Carbon13

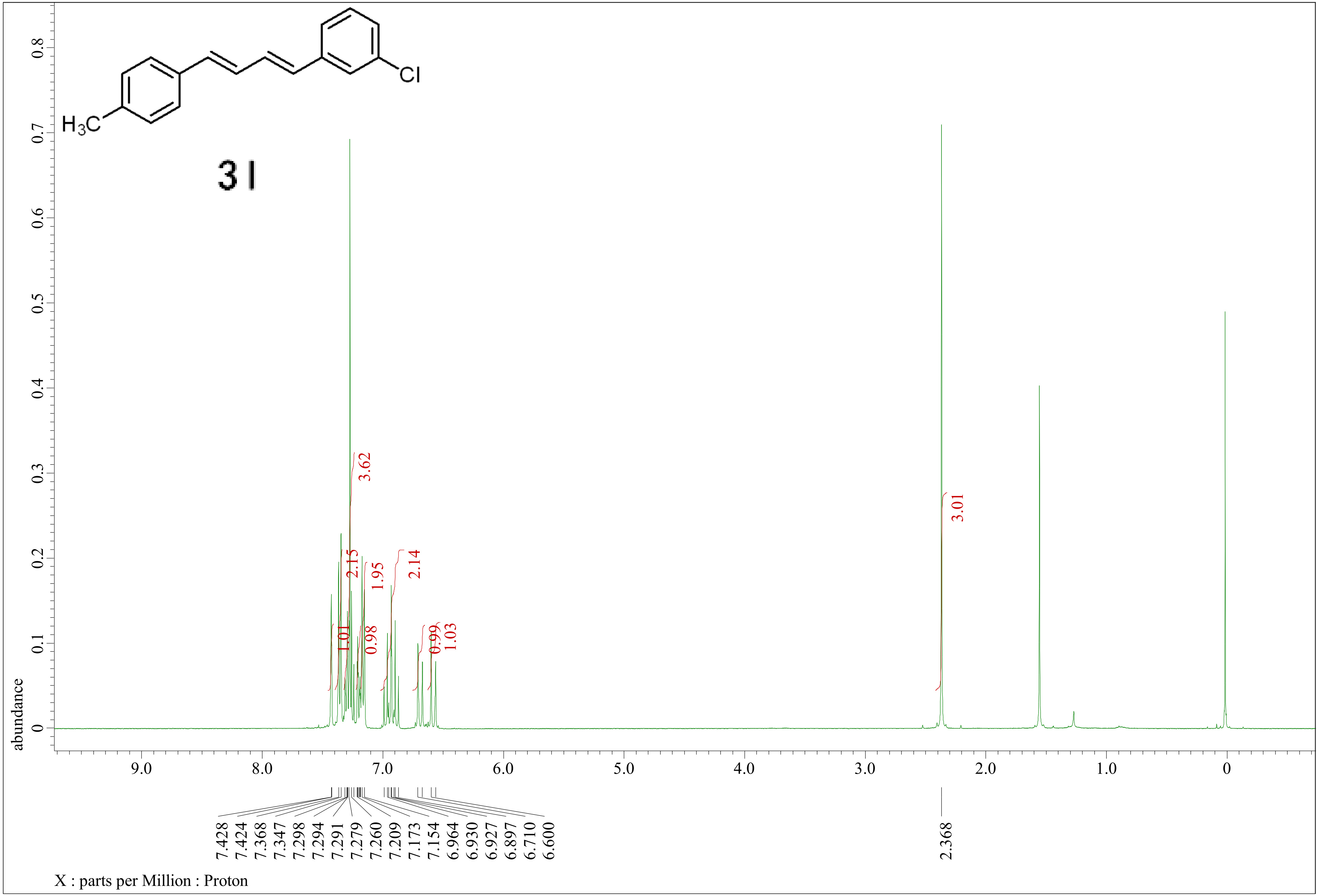

Cl
H3C
3 l
abundance
0.8
0.7
0.6
0.5
0.4
0.3
0.2
0.1
0
3.62
2.15
1.95
2.14
1.01
0.98
0.99
1.03
3.01
9.0
8.0
7.0
6.0
5.0
4.0
3.0
2.0
1.0
0
7.428
7.424
7.368
7.347
7.298
7.294
7.291
7.279
7.260
7.209
7.173
7.154
6.964
6.930
6.927
6.897
6.710
6.600
2.368
X : parts per Million : Proton

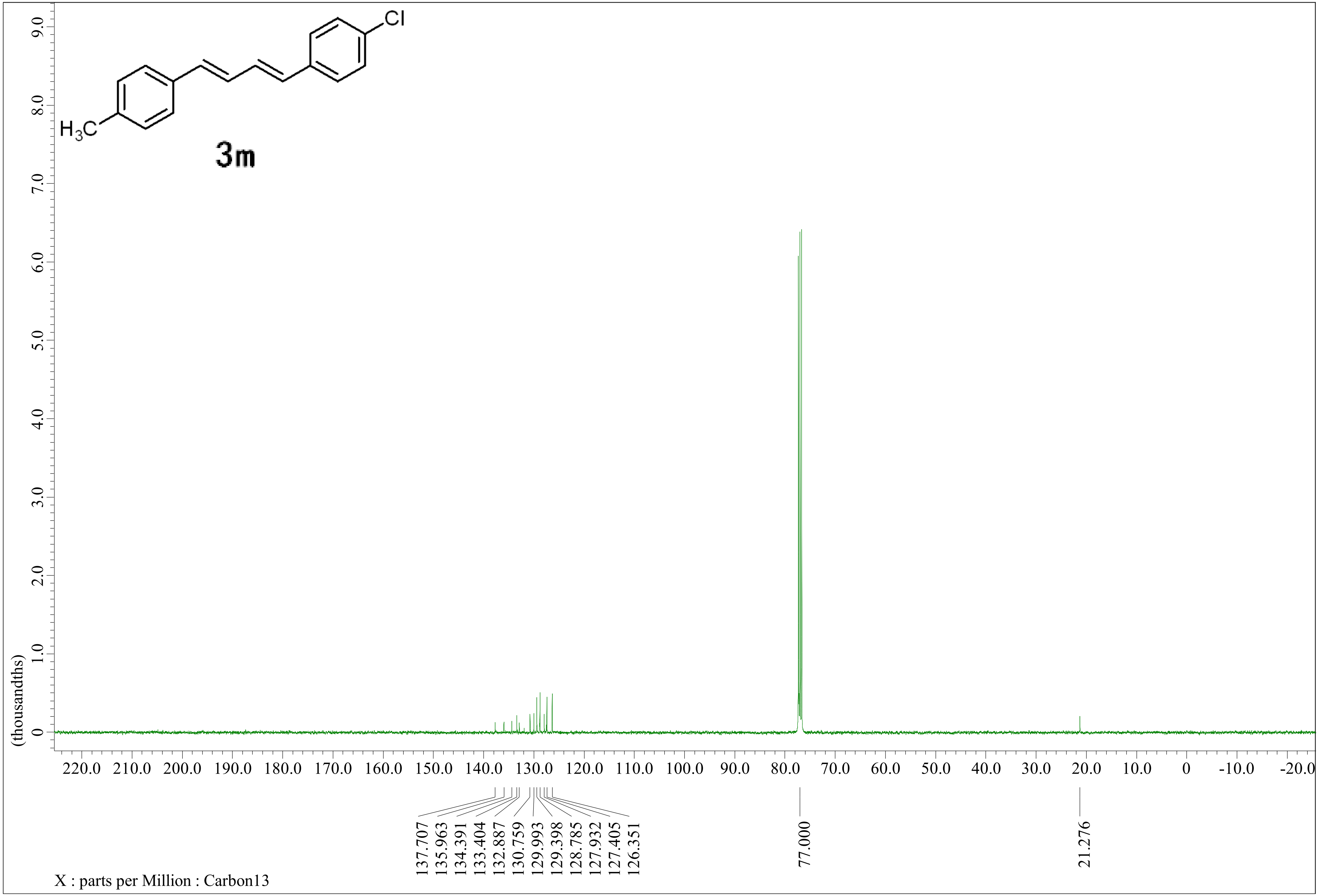
Cl
H3C
3m
(thousandths)
9.0
8.0
7.0
6.0
5.0
4.0
3.0
2.0
1.0
0
220.0 210.0 200.0 190.0 180.0 170.0 160.0 150.0 140.0 130.0 120.0 110.0 100.0 90.0 80.0 70.0 60.0 50.0 40.0 30.0 20.0 10.0 0 -10.0 -20.0
137.707
135.963
134.391
133.404
132.887
130.759
129.993
129.398
128.785
127.932
127.405
126.351
77.000
21.276
X : parts per Million : Carbon13

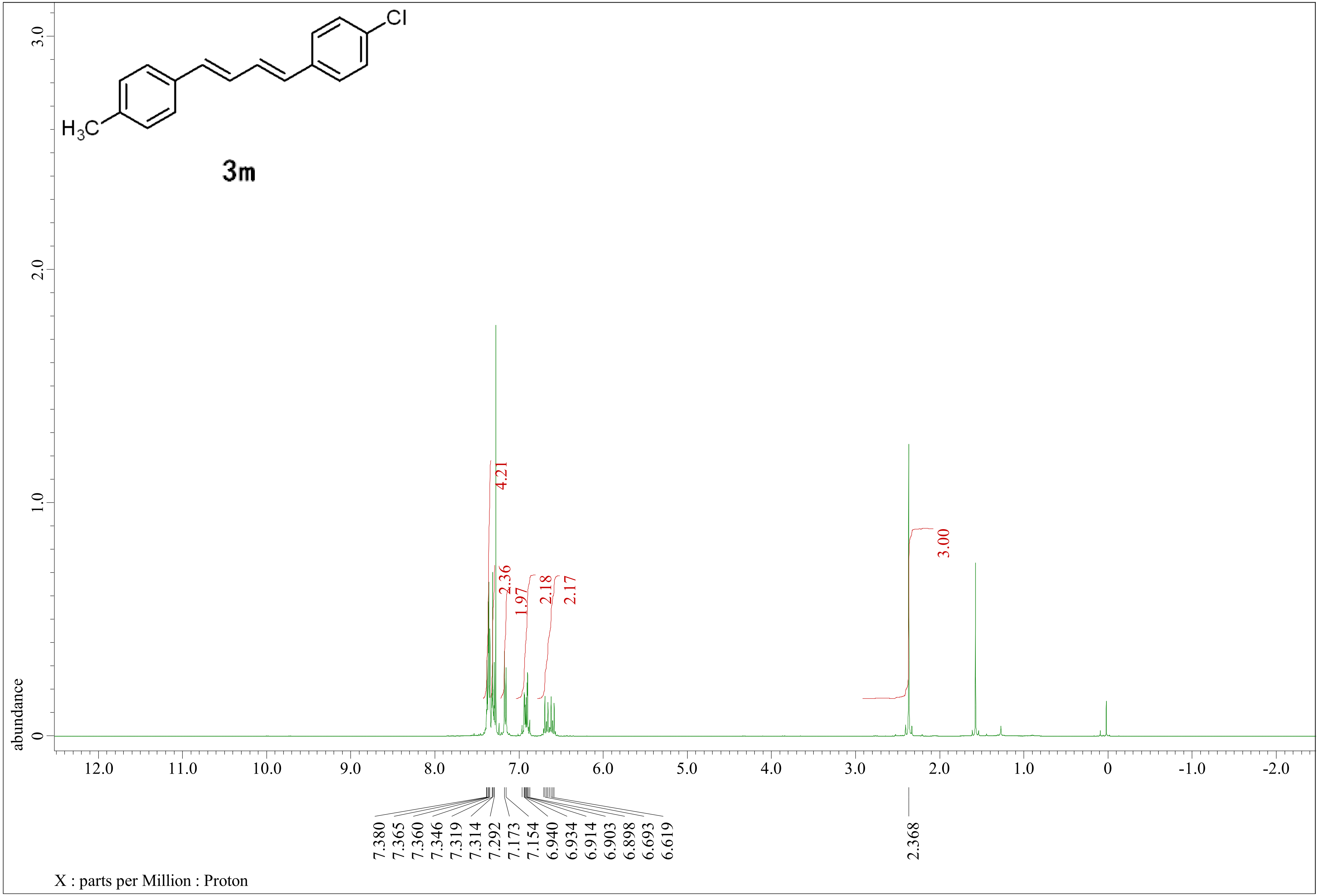

Cl
H3C
3m
abundance
3.0
2.0
1.0
0
12.0
11.0
10.0
9.0
8.0
7.0
6.0
5.0
4.0
3.0
2.0
1.0
0
-1.0
-2.0
4.21
2.36
1.97
2.18
2.17
3.00
7.380
7.365
7.360
7.346
7.319
7.314
7.292
7.173
7.154
6.940
6.934
6.914
6.903
6.898
6.693
6.619
2.368
X : parts per Million : Proton

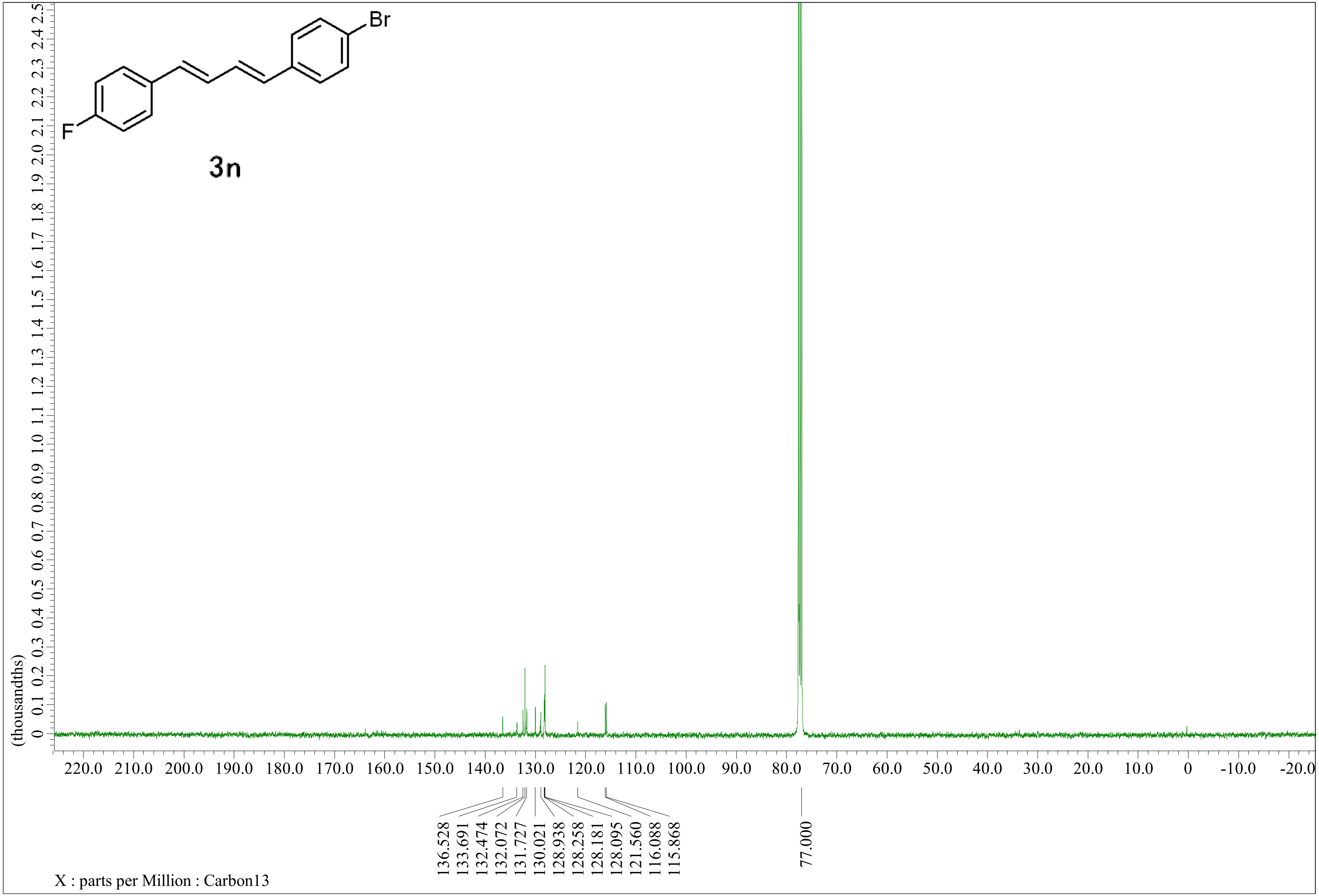

Br
F
3n
(thousandths)
0 0.1 0.2 0.3 0.4 0.5 0.6 0.7 0.8 0.9 1.0 1.1 1.2 1.3 1.4 1.5 1.6 1.7 1.8 1.9 2.0 2.1 2.2 2.3 2.4 2.5
220.0 210.0 200.0 190.0 180.0 170.0 160.0 150.0 140.0 130.0 120.0 110.0 100.0 90.0 80.0 70.0 60.0 50.0 40.0 30.0 20.0 10.0 0 -10.0 -20.0
136.528
133.691
132.474
132.072
131.727
130.021
128.938
128.258
128.181
128.095
121.560
116.088
115.868
77.000
X : parts per Million : Carbon13

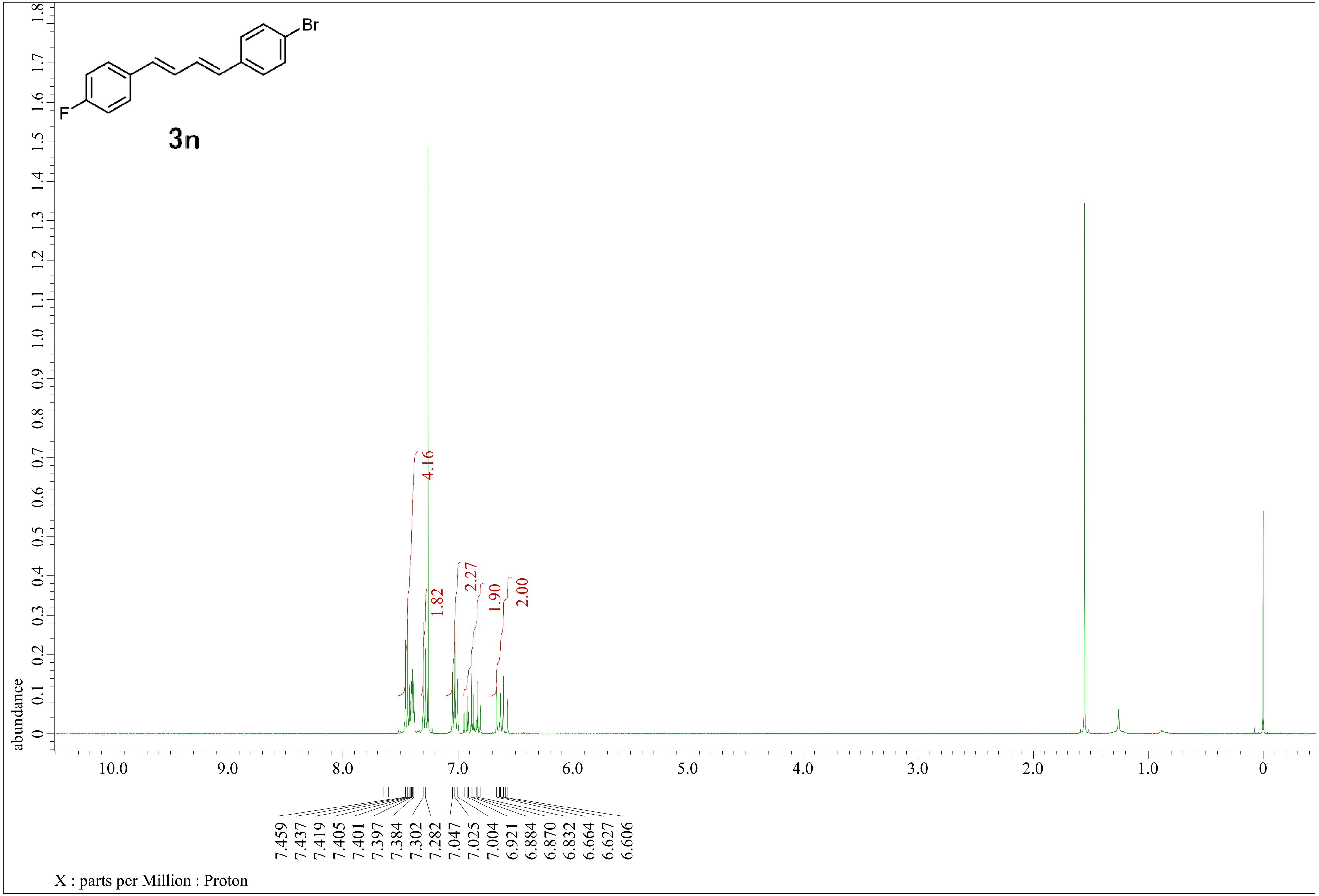

Br
F
3n
abundance
X : parts per Million : Proton
4.16
1.82
2.27
1.90
2.00
7.459
7.437
7.419
7.405
7.401
7.397
7.384
7.302
7.282
7.047
7.025
7.004
6.921
6.884
6.870
6.832
6.664
6.627
6.606
